# Kinetic and Thermodynamic Enhancement of Low-Temperature Oxygen Release from Strontium Ferrite Perovskites Modified with Ag and CeO_2_

**DOI:** 10.1021/acs.energyfuels.3c01263

**Published:** 2023-06-14

**Authors:** Alexander R. P. Harrison, Kien Y. Kwong, Yaoyao Zheng, Abhishek Balkrishna, Alice Dyson, Ewa J. Marek

**Affiliations:** † Department of Chemical Engineering and Biotechnology, University of Cambridge, Philippa Fawcett Drive, CB3 0AS Cambridge, U.K.; ‡ Department of Engineering, University of Cambridge, Trumpington Street, CB2 1PZ Cambridge, U.K.

## Abstract

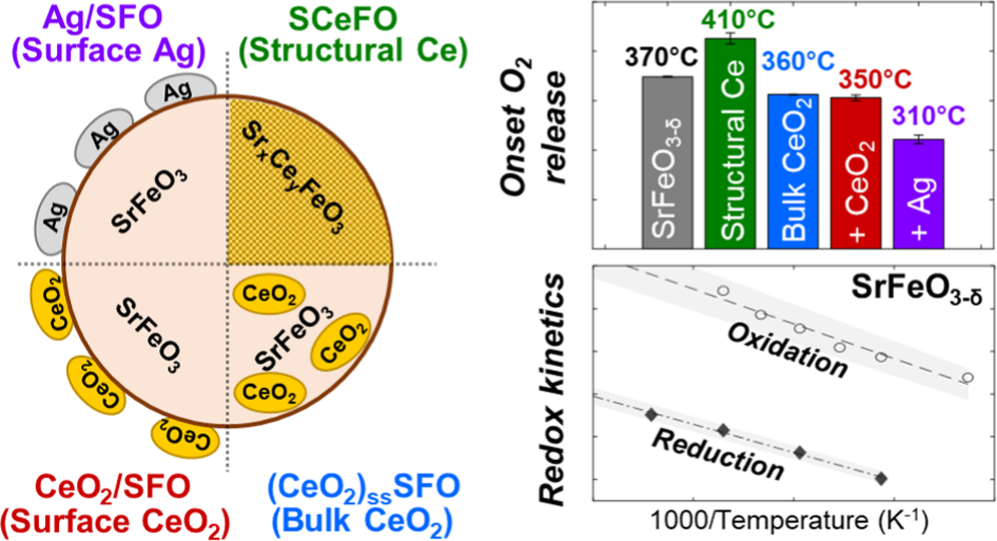

The redox behavior of the nonstoichiometric perovskite oxide SrFeO_3−δ_ modified with Ag, CeO_2_, and Ce was assessed for chemical looping air separation (CLAS) via thermogravimetric analysis and by cyclic release and uptake of O_2_ in a packed bed reactor. The results demonstrated that the addition of ∼15 wt % Ag at the surface of SrFeO_3−δ_ lowers the temperature of oxygen release in N_2_ by ∼60 °C (i.e., from 370 °C for bare SrFeO_3−δ_ to 310 °C) and more than triples the amount of oxygen released per CLAS cycle at 500 °C. Impregnation of SrFeO_3−δ_ with Ag increased the concentration of oxygen vacancies at equilibrium, lowering (3 – δ) under all investigated oxygen partial pressures. The addition of CeO_2_ at the surface or into the bulk of SrFeO_3−δ_ resulted in more modest changes, with a decrease in temperature for O_2_ release of 20–25 °C as compared to SrFeO_3−δ_ and a moderate increase in oxygen yield per reduction cycle. The apparent kinetic parameters for reduction of SrFeO_3−δ_, with Ag and CeO_2_ additives, were determined from the CLAS experiments in a packed bed reactor, giving activation energies and pre-exponential factors of *E*_a,reduction_ = 66.3 kJ mol^–1^ and *A*_reduction_ = 152 mol s^–1^ m^–3^ Pa^–1^ for SrFeO_3−δ_ impregnated with 10.7 wt % CeO_2_, 75.7 kJ mol^–1^ and 623 mol_O_2__ s^–1^ m ^–3^ Pa^–1^ for SrFeO_3−δ_ mixed with 2.5 wt % CeO_2_ in the bulk, 29.9 kJ mol^–1^ and 0.88 mol_O_2__ s^–1^ m^–3^ Pa^–1^ for Sr_0.95_Ce_0.05_FeO_3−δ_, and 69.0 kJ mol^–1^ and 278 mol_O_2__ s^–1^ m^–3^ Pa^–1^ for SrFeO_3−δ_ impregnated with 12.7 wt % Ag, respectively. Kinetics for reoxidation were much faster and were assessed for two materials with the slowest oxygen uptake, SrFeO_3−δ_, giving the activation energy *E*_a,oxidation_ = 177.1 kJ mol^–1^ and pre-exponential factor *A*_oxidation_ = 3.40 × 10^10^ mol_O_2__ s^–1^ m^–3^ Pa^–1^, and Sr_0.95_Ce_0.05_FeO_3−δ_, giving the activation energy *E*_a,oxidation_ = 64.0 kJ mol^–1^, and pre-exponential factor *A*_oxidation_ = 584 mol_O_2__ s^–1^ m^–3^ Pa^–1^.

## 1 Introduction

Air separation is a physicochemical process for producing purified oxygen and nitrogen, with oxygen used in a variety of industrial and medical applications,^1^ as well as in novel energy technologies, such as oxy-fuel combustion.^1,2^ The predominant method for industrial air separation is cryogenic distillation, which has a relatively high capital cost and energy requirements of around 200–240 kWh/t_O_2__.^3,4^ Hence, the high resulting cost of producing refined gases cryogenically limits the cost-effectiveness of technologies that utilize pure O_2_.

**Figure 1:**
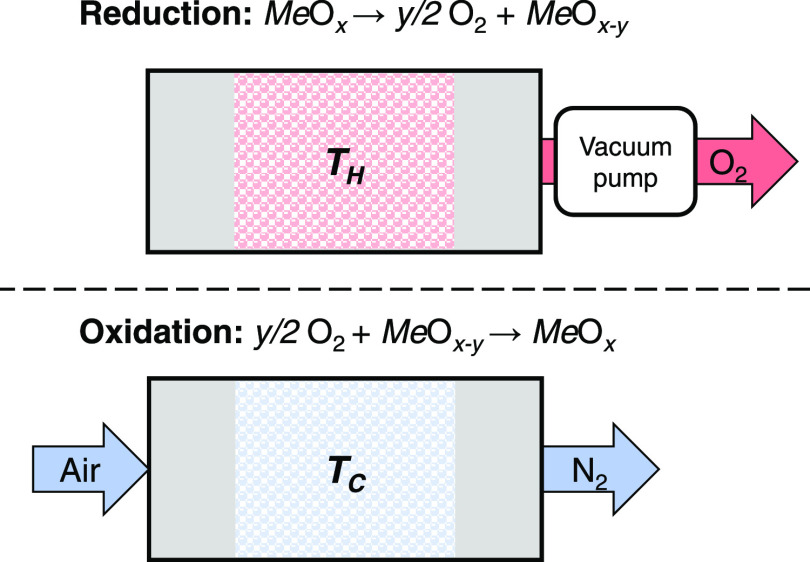
Schematic diagram showing the fundamental steps in the CLAS concept for a generic oxygen carrier MeO*_x_*. Reduction and oxidation are performed at *T*_H_ and *T*_C_, respectively (*T*_H_ > *T*_C_).

An alternative process for air separation that offers a lower specific energy requirement per unit of oxygen generated is chemical looping air separation (CLAS)^1^ with energy demand assessed to be ∼60 kWh/t_O_2__.^5^ In a CLAS system, shown schematically in Figure 1, a metal oxide, termed an oxygen carrier (OC), decomposes at a high temperature, *T*_H_, releasing oxygen. The reduced solid is easily reoxidized in air at a lower temperature, *T*_C_, closing the chemical loop and returning the OC to its original state. If the regeneration step is fast, the CLAS system can remove all O_2_ from the incoming air, producing a purified nitrogen stream, for use as a feedstock in ammonia synthesis.^6^

A class of materials of interest is nonstoichiometric perovskite oxides,^7,8^ with the general formula *AB*O_3−δ_, as they release oxygen at lower temperatures than conventional stoichiometric oxides (e.g., CuO, Fe_2_O_3_) and with faster kinetics, albeit with lower specific oxygen capacity.^9−11^ Material screening, using in silico and thermogravimetric methods, has identified strontium ferrite, SrFeO_3−δ_, as a suitable candidate material for CLAS,^12,13^ able to release oxygen in air at a lower *T*_H_ (400 °C) than most other studied materials. Particles of perovskite OC materials for CLAS can be produced via straightforward solid-state methods from oxide and carbonate precursors^14,15^ and have demonstrated stable CLAS performance with fast kinetics of oxygen release over up to 1000 redox cycles in air and N_2_.^16^ However, kinetics for oxidation of SrFeO_3−δ_ have not yet been reported.

Another benefit of perovskites, in particular, SrFeO_3−δ_, is their tuneability using relatively simple methods, e.g., by introducing bulk,^17,18^ structural,^19^ or surface^19,20^ dopants. The addition of noble metals to perovskites (Ag,^20^ Pd,^21,22^ Pt^20,22^) has been shown to aid oxygen release at lower temperatures than those for unmodified perovskites. The enhancement of oxygen release caused by the presence of noble metals on the perovskite surface is possibly caused by altering the structure of the perovskite near the metal–perovskite interface and donating electrons from the noble metal to the perovskite, reducing the energy barrier for oxygen ion transport,^20^ and/or by catalyzing the reaction of O_lattice_ ions to form gaseous O_2_ molecules and oxygen vacancies in the crystal lattice.^23,24^

The impregnation of SrFeO_3−δ_ with particles of Ag or CeO_2_ has been exploited in chemical looping catalytic systems for selective oxidation reactions,^25,26^ leading to more active OCs as compared to the unmodified SrFeO_3−δ_.^26,27^ Metal oxides, such as CeO_2_,^17,18^ CaO,^28^ or FeO*_x_*,^29,30^ introduced to the bulk of OC particles can also affect the rate of oxygen release from the perovskite, resulting in improved performance in chemical looping selective oxidation reactions. However, the effect of Ag or metal oxides as additives on altering the availability of oxygen and the rate of reduction of SrFeO_3−δ_ in chemical looping air separation has not been studied systematically.

The partial substitution of Sr and Fe with other elements to influence the specific oxygen capacity, and the minimum *T*_H_ required for oxygen release, has been widely investigated.^31,32^ For example, the partial substitution of Sr with Ce, giving Sr_1–*x*_Ce_*x*_FeO_3_, facilitates the ionic conductivity of O^2–^ through the crystal lattice, possibly by partial reduction of Ce^4+^ to Ce^3+^.^33,34^

The stoichiometry of perovskite materials at thermodynamic equilibrium, which determines the maximum oxygen capacity and chemical driving force for oxygen release, is a function of temperature and oxygen partial pressure.^35^ The effects of partial substitution of Sr in SrFeO_3_ on the thermodynamic properties of the perovskite (e.g., nonstoichiometry, 3 – δ, at equilibrium) have been investigated in experimental and theoretical studies.^36−38^ For example, Krzystowczyk et al.^37^ found that at low temperatures (≤600 °C), partial substitution of Sr with Ba or Y increased the concentration of oxygen vacancies at equilibrium, whereas doping with K or La resulted in a slight decrease. Similarly, Vieten et al.^36^ found that substitution of Sr with Mn increased the concentration of oxygen vacancies, with the extent of increase becoming greater at higher temperatures (up to 1200 °C). However, the effect of interactions between a perovskite OC and another phase (e.g., Ag or CeO_2_) on the oxygen nonstiochiometry of the SrFeO_3−δ_ perovskite at equilibrium is not well understood.

In this work, several methods of manipulating the oxygen release from SrFeO_3−δ_ were investigated using Ag, Ce, and CeO_2_ as additives with the main goal to assess whether the introduction of these three dopants affects kinetics and/or the maximum oxygen availability from SrFeO_3−δ_ at equilibrium under given pO_2_ conditions. Three methods of engineering SrFeO_3−δ_ were investigated: (1) introducing Ag or CeO_2_ to the surface of SrFeO_3_ via surface impregnation, (2) doping CeO_2_ into the bulk of SrFeO_3_, thus creating a two-phase composite of the two oxides, and (3) substituting 5 mol % Sr with Ce through structural doping to produce Sr_0.95_Ce_0.05_FeO_3−δ_. The materials prepared were then investigated via thermogravimetric analysis (TGA), and gas cycling experiments in a packed bed reactor to determine the effects of each modification method on the temperature of oxygen release and the apparent kinetics of oxygen release and reuptake in a CLAS system.

## 2 Materials and Methods

### 2.1 Material Preparation

Strontium ferrite (SrFeO_3−δ_) was prepared using a solid-state method.^14^ Stoichiometric quantities of SrCO_3_ (0.5 mol, Sigma-Aldrich, >98%) and Fe_2_O_3_ (0.25 mol, Honeywell Fluka, >99%) were mixed with ethanol (75 mL, Fisher Scientific, 99.8%) to form a homogeneous mixture. The mixture was milled using a ball mill (Pulverisette 6, Fritsch) with 13 stainless steel balls (20 mm diameter) in a stainless steel grinding jar, with 2 min of milling at 600 rpm, followed by 20 min of passive cooling between milling steps, repeated over 15 cycles. The resulting paste was dried in static air at 50 °C for 24 h, then crushed, and sieved to <355 μm. The particles were calcined in two steps of 4 and 18 h (durations chosen arbitrarily) at 1000 °C, with cooling to room temperature between the calcination steps, and then sieved to 180–355 μm.

Strontium ferrite impregnated with CeO_2_ was prepared by dissolving 0.929 g of Ce(NO_3_)_3_·6H_2_O (Acros Organics, 99.5%) in DI water, with the resulting solution then added dropwise to 2.00 g of SrFeO_3−δ_ (180–355 μm) until particles coalesced (with a volume of solution used as a proxy for the approximate volume of macropores within the material), consequently resulting in an expected loading of 9.3 wt % CeO_2_. The impregnated particles were dried at 120 °C for 12 h and then calcined at 650 °C for 5 h in static air.

Strontium ferrite with CeO_2_ mixed into the bulk was prepared by mixing 0.025 mol CeO_2_ with 0.5 mol SrCO_3_ and 0.25 mol Fe_2_O_3_ for a target loading of 4.3 wt % (5.0 mol %) CeO_2_. The sample was then milled, dried, and calcined as for SrFeO_3−δ_. Particles of CeO_2_ were prepared by slow thermal decomposition of Ce(NO_3_)_3_·6H_2_O (Acros Organics, 99.5%) at 500 °C for 4 h in air, at a heating rate of 1 °C min^–1^, as described elsewhere.^17^

Cerium-doped strontium ferrite, Sr_0.95_Ce_0.05_FeO_3-δ_, was prepared by mixing 0.025 mol CeO_2_ with 0.475 mol SrCO_3_ and 0.25 mol Fe_2_O_3_. The sample was milled, dried, and calcined at 1200 °C in two steps of 4 and 18 h, respectively, to ensure incorporation of Ce into the perovskite structure.^39^ To activate Sr_0.95_Ce_0.05_FeO_3−δ_, the particles of the synthesized material were reduced for 1.5 h in 4.87 vol % H_2_ (balance N_2_, Air Liquide) at 700 °C, then reoxidised by holding the sample at 650 °C in air for 30 min, and slowly cooled to room temperature at a ramp rate of −2 °C min^–1^ to ensure incorporation of oxygen into the perovskite structure. After the activation step, Ce remained in the perovskite structure, as shown by X-ray diffraction (XRD) characterization, as compared with CeO_2_/SFO and (CeO_2_)_ss_SFO, where XRD peaks corresponding to CeO_2_ were visible (shown in the SI, Section S1 and Figure S1).

The final sample was SrFeO_3−δ_ impregnated with Ag. Particles of silver were added to the surface of SrFeO_3−δ_ by incipient-wetness impregnation.^27^ A silver precursor solution was prepared by dissolving AgNO_3_ (Alfa Aesar, >99.8%) in deionized water. The resulting solution was added dropwise to 2.00 g of particles of SrFeO_3_ (180–355 μm), for a target loading of 15 wt % Ag, followed by drying and calcining the same as for impregnation of CeO_2_.

The samples prepared and abbreviations used hereafter are summarized in Table 1.

**Table 1:** Summary of Samples Prepared, Abbreviations Used, and Loadings of Ag or CeO_2_ as Determined from ICP-AES Measurements

sample	abbreviated name	target loading	measured loading
strontium ferrite, SrFeO_3−δ_	SFO		
ceria-impregnated strontium ferrite, CeO_2_/SrFeO_3−δ_	CeO_2_/SFO	9.3 wt % CeO_2_	10.6 wt % CeO_2_
strontium ferrite with ceria added to bulk, (CeO_2_)_ss_SrFeO_3−δ_	(CeO_2_)_ss_SFO	4.3 wt % CeO_2_	2.5 wt % CeO_2_
cerium-doped strontium ferrite, Sr_0.95_Ce_0.05_FeO_3−δ_	SCeFO		
silver-impregnated strontium ferrite, Ag/SrFeO_3−δ_	Ag/SFO	15.0 wt % Ag	12.7 wt % Ag

### 2.2 Material Characterization

X-ray diffraction (XRD) measurements of powder samples were taken using a Bruker D8 Discover powder X-ray diffractometer using Cu Kα radiation over the angular range 2θ = 20–80° with a step size of 0.05° and a step time of 2 s. The phase composition was estimated using Profex software,^40^ with reference structures from the ICSD database.^41^ ICSD collection codes used are given in the SI, Table S1. The compositions of materials were further estimated using inductively coupled plasma atomic emission spectroscopy (ICP-AES). The results of the XRD and ICP-AES characterization of prepared materials used are given in Section S1 of the SI, Figure S1 and Tables S2–S4, showing that the OC materials were predominantly composed of the cubic SrFeO_3_ perovskite phase (>95 wt %), with the amounts of Ag and CeO_2_ phases close to the expected loadings. Spent samples of OC materials after 250 CLAS cycles were characterized by XRD, as shown in Figure S2, confirming that the SrFeO_3_ phase with perovskite structure remained the predominant phase. For SCeFO, incorporation of Ce into the perovskite structure was confirmed by a characteristic shift of the XRD peak at 2θ ≈ 47°, as shown in Figure S3.

Scanning electron microscopy with energy-dispersive X-ray spectroscopy (SEM-EDS) images were acquired using a Tescan MIRA3 FEG-SEM. Samples were prepared using a thin surface coating of Pt (∼20 nm) to mitigate the effect of surface charging. The distribution of sizes of Ag particles was estimated from SEM images using ImageJ software,^42^ demonstrating agglomeration of Ag after 250 cycles of CLAS performed at 500–600 °C (see Figure S4 in the SI). Of the OC materials containing Ce, CeO_2_/SFO and (CeO_2_)_ss_SFO showed similar surface morphology to Ag/SFO (given in the SI, Figures S5 and S6), whereas the sample of SCeFO (Figure S7) was significantly less porous. The low porosity of SCeFO was caused by calcination at a higher temperature than the other samples, 1200 °C rather than 1000 °C, which was applied to ensure incorporation of Ce into the perovskite structure.

### 2.3 Thermogravimetric Analysis

Thermogravimetric analysis (TGA) of samples was performed using a Metter Toledo TGA/DSC 1 analyzer with a horizontally oriented microbalance and the reactive gas delivered over the sample via a capillary tube, with the flow rate of the reactive gas set to 50 mL min^–1^. An alumina crucible was loaded with 20–50 mg of particulate sample and placed on the balance pan. For temperature-programmed reduction and oxidation experiments, the sample was heated from 25 to 900 °C (TPR) at a heating rate of 10 °C min^–1^ in a flow of N_2_ and then cooled from 900 to 25 °C in air (TPO). The chamber and balance were continuously purged with a protective flow of N_2_, each with a flow rate of 50 mL min^–1^. For isothermal gas-switching experiments, the sample was heated from 25 °C to a given temperature in the range of 400–600 °C in air. The gas flow was switched to N_2_ for 45 min to reduce the sample, then switched back to air for 15 min to reoxidize the sample. Three cycles were performed for each temperature. All gases were BOC, >99.998%, with the total flow rate of gases through the chamber set to 150 mL min^–1^ (NTP).

Initial oxygen nonstoichiometry at room temperature of the SFO and Ag/SFO materials was estimated by heating the OC material in 5 vol % H_2_ (balance N_2_) from 25 to 900 °C. From the resulting TPR curve, a marked change in the gradient was observed corresponding to the reduction of brownmillerite SrFeO_2.5_.^14^ Hence, by taking the stoichiometry of SrFeO_3−δ_ to be equal to (3 – δ) = 2.5 at the point where the change in gradient was observed and assuming that the mass of Ag in the Ag/SFO sample remained constant during reduction, the initial values of (3 – δ_0_) were calculated to be 2.82 ± 0.026 for SFO and 2.83 ± 0.003 for Ag/SFO. To estimate the pO_2_–*T*–δ characteristics for SFO and Ag/SFO, a sample of the OC material was placed in the TGA instrument, with purge, protective, and reactive streams supplied using the same air–N_2_ blend, mixed to obtain target pO_2_ in the range of 10^–5^–0.21 atm. The required ratio of air and N_2_ flow rates was set using rotameters, with the total flow to the TGA instrument kept at 150 mL min^–1^ for all experiments. The experiment started by ramping the temperature to a set-point in the range of 500–600 °C, followed by a 3 h isothermal hold to allow the investigated sample to reach equilibrium with the flowing gas. The pO_2_ of the gas stream leaving the TGA instrument was measured during experiments using an ABB EL3020 analyzer fitted with a Magnos 206 paramagnetic oxygen sensor. Reference *T–*δ_eq_ curves were calculated with FactSage 8.2 software^43^ using thermodynamic data for SrFeO_3-δ_ from Preis.^44^

### 2.4 Experiments in the Packed Bed Reactor

Experiments were performed using a tubular packed bed microreactor, with a schematic of the reactor given in the SI, Section S2, Figure S8. The reactor was constructed from a stainless steel tube with an internal diameter of 4.8 mm and a length of 8.0 cm. The packed bed, composed of a preheating section of 0.60 g of α-Al_2_O_3_, 150–355 μm, and an active bed of 0.40 g of the SrFeO_3−δ_-based oxygen carrier, 180–355 μm, was placed inside the reactor and secured using alumina wool. The feed to the reactor was controlled using two solenoid flipper valves (Bürkert Type 6124-2/2 way) connected to air and N_2_ cylinders (both BOC, >99.998%). The flow rate of gases to the reactor was controlled using an orifice place (i.d. 0.61 mm). Flow through the reactor was set to 750 mL min^–1^ (NTP) by adjusting the inlet pressure upstream of the orifice plate and checking the flow rate with an Agilent ADM digital flowmeter. The reactor tube was heated using a high-temperature heating tape (Briskheat TBIH102), controlled with a K-type thermocouple (1.5 mm diameter) placed at the center of the packed bed. The oxygen content of the outlet gas was measured using a Bosch 4.9 LSU wideband universal exhaust gas sensor (UEGO) connected to a control unit (Cambustion Ltd.). Oxygen partial pressure was measured at a nominal sample rate of 50 s^–1^, giving a temporal resolution of 20 ms. Experiments were carried out over the temperature range of 450–600 °C, performing redox cycles, with each cycle comprising 30 s oxidation in air, followed by 30 s reduction in N_2_. Given only the initial rate of reduction, when the material is the furthest from equilibrium, is used to determine kinetic parameters, 30 s reduction cycles were adequate for packed bed experiments, as opposed to 45 min reduction used in TGA experiments.

**Figure 2:**
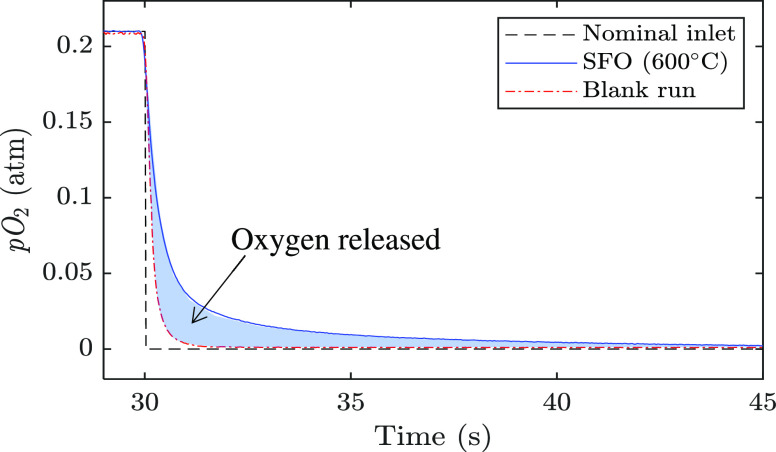
Comparison between the blank run, reduction of SFO at 600 °C, and the nominal inlet feed with the lag time subtracted. Oxygen released from SFO is determined from the area between the SFO and blank curves.

The dead time of the reactor, *t*_dead_, defined as the time interval between sending an electronic signal to the solenoid valves to change feed gas from air to nitrogen and the smoothed sensor voltage decreasing to 95% of the initial value was estimated to be 0.17 ± 0.01 s for an inert bed of 1.00 g of α-Al_2_O_3_ (i.e., 0.5% of reduction or oxidation cycle duration). The *t*_dead_ value was subtracted from the nominal start time of reduction or oxidation to account for the lag between the opening of a solenoid valve and the gas arrival at the oxygen sensor. To account for gas mixing and dispersion within the reactor, a blank run was generated by switching between air and N_2_ over an inert bed of 1.00 g of α-Al_2_O_3_, again subtracting the *t*_dead_ to ensure all runs were in sync with the timing of the opening of solenoid valves. The blank run was then subtracted from measurements with active material, compensating for dispersion and mixing within the bed during gas CLAS experiments. A comparison between the blank run and the measurement for an active bed is shown in Figure 2.

The instantaneous specific rate of oxygen release, *r* (μmol_O_2__ s^–1^ g_OC_^–1^), was estimated using
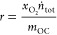
1a
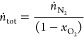
1bwhere *x*_O_2__ is the mole fraction of oxygen measured in the exhaust stream, *ṅ*_tot_ is the total outlet molar flow rate (μmol s^–1^), *ṅ*_N_2__ is the inlet molar flow rate of N_2_ (658 μmol s^–1^, measured at room temperature; assumed to be constant), and *m*_OC_ is the mass of the OC sample (g). The total amount of oxygen released or taken up by the material over each reduction or oxidation cycle was then estimated by integrating the instantaneous rate over the cycle duration.

To estimate the apparent kinetics of oxygen release from measurements taken using the packed bed reactor, the method employed by Görke et al.^16^ was used to derive an expression for the first-order rate constant, *k*, with respect to the maximum rate of oxygen release for each CLAS cycle, accounting for changing the gas velocity along the length of the packed bed. A full derivation of the rate expression used is given in the SI, Section S3, eqs S3–S5.

Instantaneously after the gas switch from air to N_2_, the material was in a fully oxidized state (i.e., conversion ∼0), and the greatest pO_2_ in the outlet gas resulting from oxygen release from the OC material was observed. Therefore, the maximum value of pO_2_ after gas switching was used to estimate the rate constant, *k*.

The variation of the rate constant, *k*, with temperature was described using the Arrhenius law
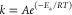
2a
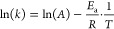
2bwhere *E*_a_ is the apparent activation energy (kJ mol^–1^) and *A* is a pre-exponential factor (mol_O_2__ s^–1^ m^–3^ Pa^–1^).

To characterize the influence of random errors from experimental measurements on kinetic parameters, values of *E*_a_ and *A* were estimated by fitting eq 2a directly to the experimentally determined values of *k* using nonlinear regression and by fitting eq 2b to the experimentally determined values of *k* using linear regression. A statistically significant difference between the two regression methods indicates that the resulting values of *A* and *E*_a_ were considerably affected by the experimental arrangement^45,46^ and, hence, do not accurately reflect the intrinsic kinetics of oxygen release.

The reoxidation of SrFeO_3−δ_-based OC materials with air at >500 °C was previously shown to be very fast, making attempts to assess kinetic parameters difficult.^16^ Additionally, here, during reoxidation in air, exothermic effects resulted in a considerable increase in the bed temperature, noticeably above the controller set-point (up to ∼15 °C for the most active samples). To limit the temperature increase and the extent of O_2_ consumption, the mass of OC materials in the bed was decreased from 0.4 to 0.1 g and the redox procedure was changed from 30 to 120 s of reduction in N_2_ at 475–600 °C, followed by 120 s reoxidation in 5.05 vol % O_2_ (balance N_2_, BOC).

## 3 Results

### 3.1 Thermogravimetric Analysis

**Figure 3:**
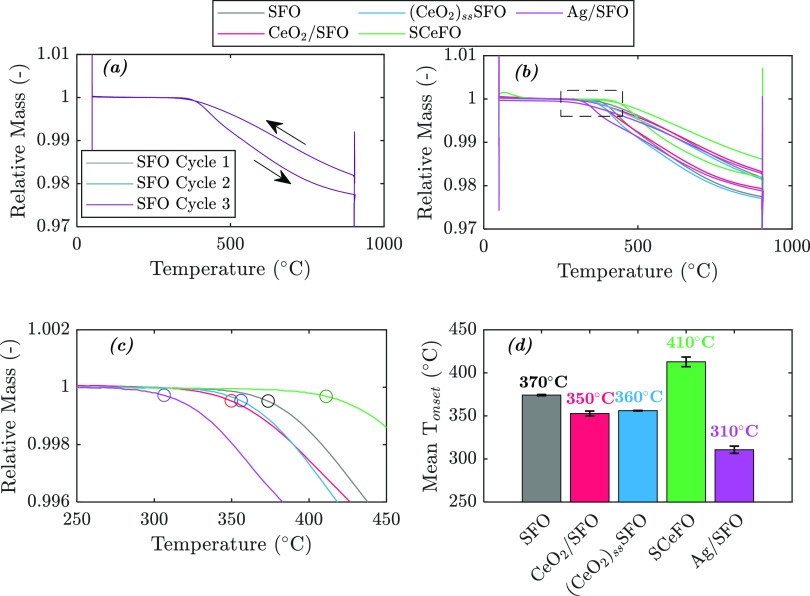
(a) Three reduction–oxidation cycles for SFO, showing consistent mass change over each TPR-TPO cycle; (b) comparison of the second TPR-TPO cycle for SFO, CeO_2_/SFO, (CeO_2_)_ss_SFO, SCeFO, and Ag/SFO; (c) details from the TPR step in (b) (dashed box), showing *T*_onset_ (indicated by circled points) for each sample; and (d) mean value of *T*_onset_ for each sample over three TPR-TPO cycles (error bars show standard deviation over three cycles). Masses were normalized with respect to the mass at the start of TPR cycles.

The reduction and oxidation behavior of the prepared materials was determined via temperature-programmed reduction in N_2_ (TPR) and oxidation in air (TPO). All samples showed a consistent mass change over the three cycles, indicating that the initial oxygen nonstoichiometry of SrFeO_3−δ_ fully regenerated after reoxidation (shown in Figure 3a for SFO and Figure S9 in the SI for all samples). Particles of CeO_2_ were also analyzed using TPR-TPO; however, the results given in the SI, Figure S10, showed that CeO_2_ on its own was practically inactive. Given that oxygen release from CeO_2_ was negligible during TPR-TPO, differences in oxygen release between the SFO, CeO_2_/SFO, and (CeO_2_)_ss_SFO samples were ascribed solely to the interaction between SrFeO_3−δ_ and CeO_2_.^47,48^ For each sample, the starting temperature of reduction in N_2_, *T*_onset_, was estimated using Matlab function “findchangepts” by identifying the point with the greatest change in gradient in the mass loss (Figure 3b,c). Impregnation with Ag on the surface of SFO was found to have the largest influence on *T*_onset_ of the samples tested, showing a decrease of 60 ± 4 °C, as compared to SFO (Figure 3d). A lower *T*_onset_ is consistent with previous studies,^27^ investigating the release of oxygen from Ag/SrFeO_3−δ_ heated in air.

Addition of CeO_2_ on the surface or in the bulk of SFO lowered the *T*_onset_ by around 20 ± 3 °C and 10 ± 0.3 °C for CeO_2_/SFO and (CeO_2_)_ss_SFO, respectively. The decrease in *T*_onset_ for (CeO_2_)_ss_SFO and CeO_2_/SFO is consistent with the literature,^17^ with previous studies reporting a decrease in *T*_onset_ for (CeO_2_)_ss_SFO and CeO_2_/SFO when reduced in H_2_ during TPR. The TPR-TPO curve for SCeFO shows a higher *T*_onset_ and less total oxygen release at 900 °C than the unmodified sample of SFO, suggesting that the structural modification by partial substitution of Sr with 5 atom % Ce might inhibit oxygen release from the perovskite lattice.

**Figure 4:**
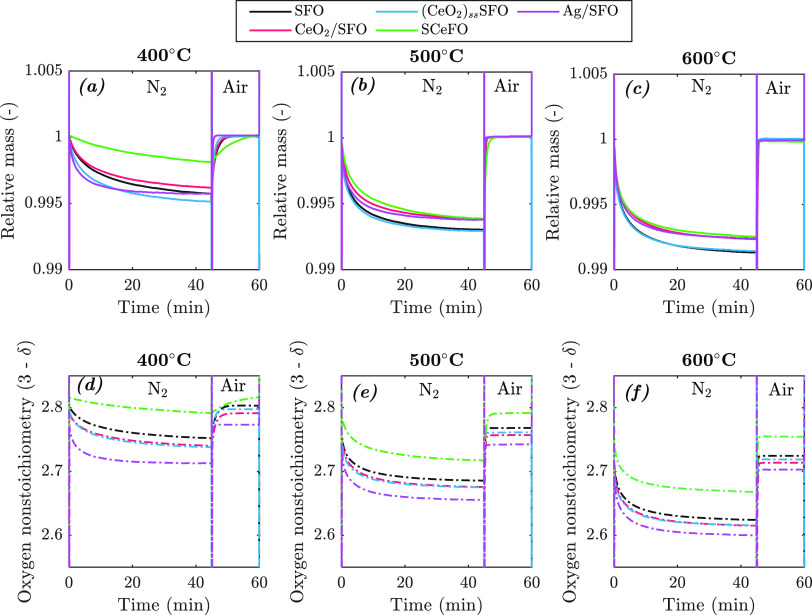
Isothermal gas cycling over SFO, CeO2/SFO, (CeO2)ssSFO, SCeFO, and Ag/SFO at 400–600 °C, with vertical lines indicating gas switching between N_2_ and air. The second of the three redox cycles is shown for all samples and temperatures. Masses normalized with respect to mass of sample in equilibrium with air at each temperature. Solid lines indicate relative mass (a–c), and dash-dot lines indicate estimated oxygen stoichiometry (d–f).

Isothermal TGA measurements over the temperature range of 400–600 °C, shown in Figure 4 with overlapping results for CLAS cycles, demonstrate that, besides the *T*_onset_, modification of SFO affects the rate of oxygen release. At 400 °C, SFO, CeO_2_/SFO, and (CeO_2_)_ss_SFO all released oxygen relatively slowly with decreasing rate of release, reaching a plateau after around 40 min. The Ag/SFO material reduced rapidly at 400 °C in N_2_, reaching 95% of the final mass change within ∼15 min. Contrastingly, SCeFO reduced at the slowest rate. Given that 400 °C is below the *T*_onset_ for SCeFO, the limited oxygen release was unsurprising. At higher temperatures (≥500 °C), the differences between samples gradually diminished, with results for SFO and (CeO_2_)_ss_SFO almost overlapping at 500 and 600 °C (shown in Figure 4b,c). The curves corresponding to the mass change for Ag/SFO, CeO_2_/SFO, and SCeFO also overlapped at 600 °C (shown in Figure 4c), with similar total mass change and rate of oxygen release. For all samples, the rate of reoxidation in air was much faster than the rate of preceding reduction. At 400 °C, Ag/SFO fully reoxidized within ∼1.5 min; SFO, CeO_2_/SFO, and (CeO_2_)_ss_SFO required about twice that time, and SCeFO—being the slowest sample—required ∼13 min. However, when the temperature was increased (≥500 °C), all samples reoxidized rapidly, in less than 1 min.

Figure 4d–f presents the change in oxygen nonstoichiometry of SrFeO_3−δ_, i.e., (3 – δ), during the isothermal CLAS cycle, demonstrating the effect of O_2_ release, shown in Figure 4a–c, on δ. The starting value of (3 – δ) for SFO at 25 °C was 2.82 and 2.83 for Ag/SFO, as determined from a separate experiment, performing TPR in H_2_ (with the methodology described in the SI, Section S4, and results shown in Figure S11). Calculations assumed that the mass of CeO_2_ in CeO_2_/SFO and (CeO_2_)_ss_SFO (taken from the ICP measurements, Table S4 in the SI) did not change over time—justified as CeO_2_ was not active during reduction in N_2_, as shown in Figure S10. Given the difficulties in estimating initial (3 – δ) for samples containing CeO_2_, due to the partial reduction of CeO_2_ under TPR in H_2_, the initial stoichiometry for samples CeO_2_/SFO, (CeO_2_)_ss_SFO, and SCeFO was estimated to be ∼2.82, as that for SFO.

The final oxygen nonstoichiometry during reduction of all samples over the temperature range of 400–600 °C (Figure 4) exhibited the same trend as *T*_onset_, with the extent of reduction following the order Ag/SFO > (CeO_2_)_ss_SFO ≈ CeO_2_/SFO > SFO > SCeFO. The findings from the isothermal experiments are therefore consistent with the observation from TPR-TPO that Ag enhances the oxygen release from SrFeO_3−δ_ to the greatest extent, CeO_2_ somewhat enhances it, and structurally doped Ce inhibits the oxygen release, as compared to SrFeO_3−δ_.

**Figure 5:**
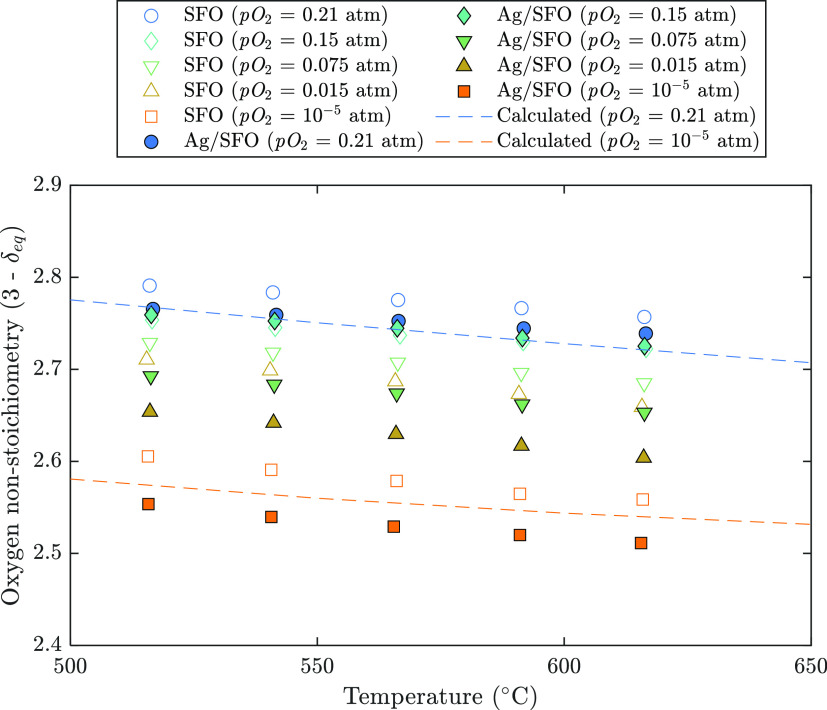
Change in oxygen nonstoichiometry with temperature for SFO (hollow symbols) and Ag/SFO (filled symbols) in various pO_2_ atmospheres. Dashed lines indicate calculated values of (3 – δ_eq_) for SrFeO_3−δ_ as a function of *T* using reference thermodynamic parameters from ref (44).

Equilibrium oxygen nonstoichiometry, (3 – δ_eq_), for SFO and Ag/SFO at varied temperatures and pO_2_ values was estimated from TGA measurements of the material exposed to a given target pO_2_ for 3 h at 25 °C increments over the range of 500–600 °C, and the results are presented in Figure 5. Curves showing δ_eq_ as a function of *T* for SrFeO_3−δ_ at pO_2_ = 0.21 and 10^–5^ atm were plotted for comparison using reference thermodynamic data for SrFeO_3-δ_ from Preis.^44^ Raw TGA curves from Figure 5 are also given in the SI, Figure S12.

The resulting values of (3 – δ_eq_) for SFO estimated here show reasonable agreement with published values of (3 – δ_eq_) for high pO_2_ values, as shown in the SI, Figure S13. At pO_2_ ≤ 0.075 atm, the values of (3 – δ_eq_) obtained here were consistently higher than those reported from TGA measurements by Ikeda et al.^49^ and Krzystowczyk et al.^37^ but lower than the TGA results from Görke et al.^16^ and Vieten et al.^36^ Additionally, Starkov et al.^50^ estimated the pO_2_–*T*–δ_eq_ characteristics for SrFeO_3−δ_ by measuring the total uptake and release of oxygen from a packed bed of SFO, giving somewhat higher values of (3 – δ_eq_) at all temperatures than those reported here, apart from at pO_2_ = 10^–5^ atm, where their estimated (3 – δ_eq_) was ∼2.5 (i.e., indicating phase transition to brownmillerite SrFeO_2.5_) at all temperatures investigated.

Comparing the obtained values for (3 – δ_eq_) for SFO and Ag/SFO in Figure 5, in most cases, Ag/SFO equilibrated at lower (3 – δ_eq_) than SFO, with a difference becoming more pronounced at lower values of pO_2_ (<0.15 atm). Hence, the presence of Ag on the surface of SrFeO_3−δ_ resulted in a structure with a higher concentration of oxygen vacancies under given pO_2_–*T* conditions.

### 3.2 Experiments in the Packed Bed Reactor

**Figure 6:**
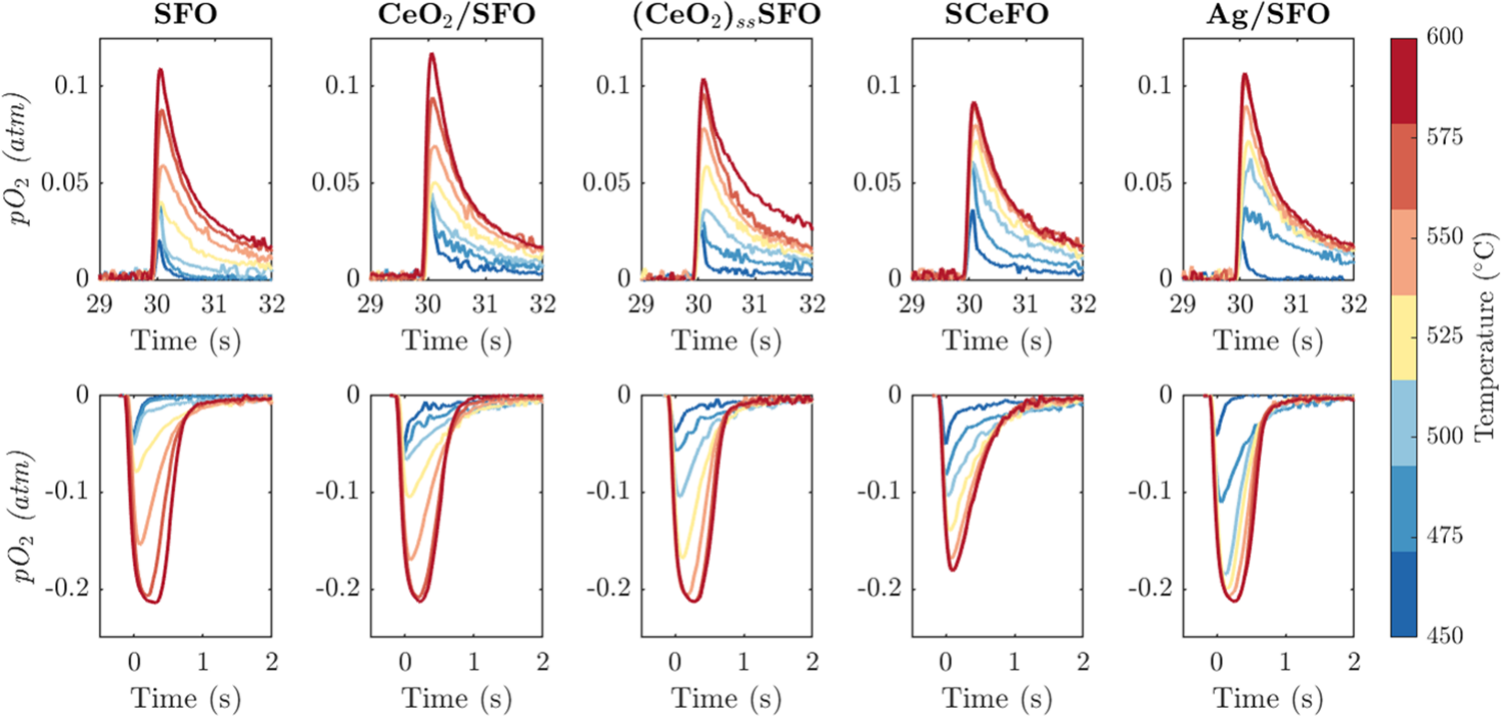
Redox cycling of OC materials in a packed bed reactor under CLAS conditions, showing oxygen release during reduction (top row) and oxygen uptake during reoxidation (bottom row) of oxygen carrier materials. In all cases, the 25th of 46–49 performed cycles is shown.

The reduction and reoxidation profiles for the oxygen carrier materials undergoing CLAS cycling in air and N_2_ are shown in Figure 6. For all samples, a sharp, asymmetric peak in the concentration of oxygen measured at the outlet of the reactor was observed following gas switching from air to nitrogen, thus indicating initially rapid, then gradually decaying, oxygen release. When the feed gas was switched back to air, all samples reoxidized rapidly, with the observed oxygen uptake taking less than 5 s. For SFO, the measured rates of oxygen release and uptake are in good agreement with previously reported results.^16^

For all measurements, excluding SCeFO, the averaged oxygen balance over each cycle, assessed by comparing the amount of oxygen released to the amount of oxygen absorbed during reduction and reoxidation, was >90%. The sample of SrFeO_3−δ_ structurally doped with Ce, SCeFO, showed an oxygen balance of only ∼80%, with a greater extent of measured oxygen release than reuptake. The observed discrepancy for SCeFO might be caused by relatively slow reoxidation, resulting in pO_2_ deviations below the detection threshold of the oxygen sensor (pO_2_ ≈ 100 Pa), giving a systematic underestimate of oxygen uptake over a full oxidation cycle.

**Figure 7:**
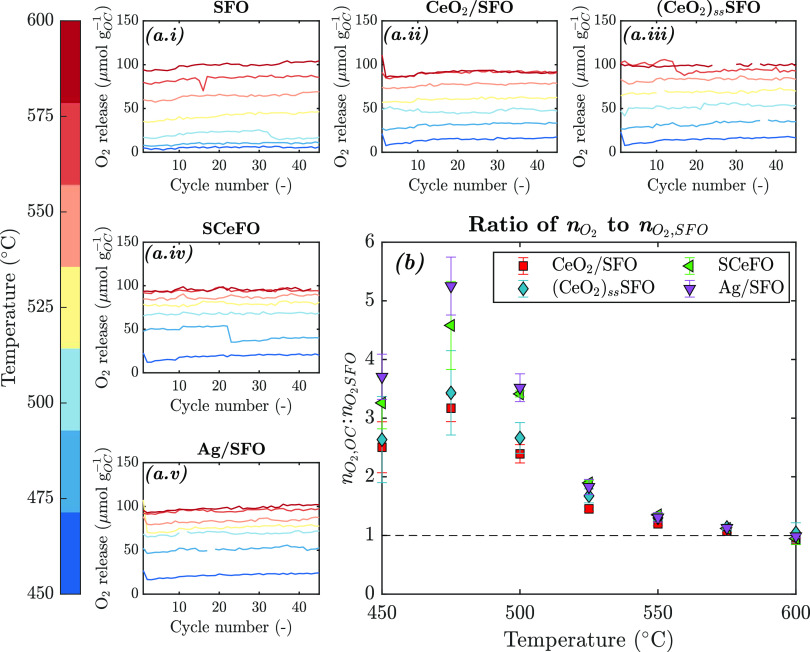
(a (i–v)) Oxygen release from SFO, CeO_2_/SFO, (CeO_2_)_ss_SFO, and Ag/SFO oxygen carrier materials in packed bed and (b) ratio of moles of oxygen released per cycle from modified oxygen carriers (n_O_2__) to moles of oxygen released from SFO (n_O_2_,SFO_). Markers indicate the average over 45–49 cycles, and error bars indicate the standard deviation over 45–49 cycles. Gaps indicate the removal of numerical artifacts, which resulted from imperfect alignment between blank and measured curves.

All samples showed stable oxygen release and uptake over the course of 46–49 CLAS cycles, as presented in Figure 7a, demonstrating that redox cycling was fully reversible with no changes in the oxygen storage capacity and confirming that SFO is able to stably release and uptake oxygen over multiple redox cycles,^16^ including when modified with CeO_2_ or Ag.

Ratios of the amount of oxygen released from modified oxygen carriers, *n*_O_2__, to the oxygen released from SFO, *n*_O_2_,SFO_, at each temperature are shown in Figure 7b. At low temperatures (<550 °C), CeO_2_/gSFO, (CeO_2_)_ss_SFO, SCeFO, and Ag/SFO all showed a marked increase in oxygen released per cycle. At all temperatures, Ag/SFO showed the greatest increase in total oxygen release, with Ag/SFO releasing approximately five times more oxygen per cycle at 475 °C. Interestingly, despite showing an increase in *T*_onset_ as compared to SFO, the sample of SCeFO released markedly more oxygen per cycle than the SFO and CeO_2_/SFO, as can be observed for each temperature point in Figure 7b.

For all samples, the oxygen released at 600 °C was approximately the same and equal to ∼95 μmol g_OC_^–1^ per cycle. The estimated rate of external mass transfer from the surface of the particles to the flowing N_2_ was estimated to be ∼50 mol s^–1^ g_OC_^–1^ (as described in the SI, Section S5), much larger than the rate of reduction of oxides. However, at 600 °C, the rate-limiting step for oxygen release might have changed from being limited by reaction kinetics to internal mass transfer as a result of diffusion within the pores of the particles of OC materials. An estimation of the Thiele modulus, ϕ, describing the relative influence of reaction kinetics and internal diffusion within pores, is given in the SI, Section S5 and Figure S14a, with approximate values of ϕ = ∼0.44–0.77 over the range of 475–600 °C, confirming the relatively low influence of internal mass transfer. For reoxidation, internal diffusion was found to limit the rate of reaction at all temperatures, with approximate values of ϕ = ∼2.5–8.2 over the range 475–600 °C, shown in Figure S14b. Hence, the estimated rate constants were recalculated to account for the effect of internal mass transport, as described in the SI, eq S14, with the modified rate constants used for estimating kinetic parameters.

The rate of reaction can also be limited by the solid and gas phases approaching thermodynamic equilibrium along the length of the packed bed, i.e., if the velocity of the reaction front is of the same order as the superficial velocity of gas traveling through the bed.^16^ As shown in the SI, Figure S15, equilibrium limitation was found to be insignificant for reduction of all materials but to some degree affected the observed rate of oxygen uptake.

## 4 Discussion

### 4.1 Estimation of Apparent Kinetic Parameters

#### 4.1.1 Kinetics of Oxygen Release

**Figure 8:**
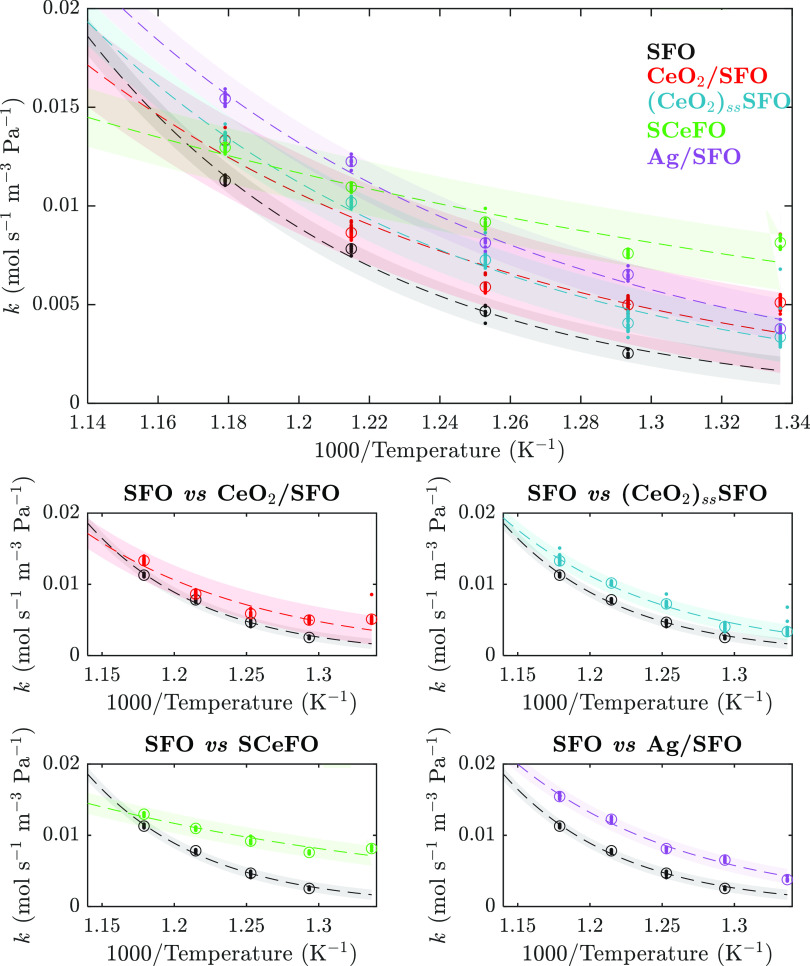
Estimated rate constant, *k*, plotted against inverse temperature for SFO over the range of 500–575 °C and for CeO_2_/SFO, (CeO_2_)_ss_SFO, and Ag/SFO over the range of 475–575 °C. Points indicate the experimental measurements from each cycle, circles indicate the average over cycles, dashed lines indicate the fitted exponential curves (eq 2a), and shaded regions indicate the 95% prediction bands. Subplots show a pairwise comparison of each modified OC sample with SFO.

The values of kinetic parameters reported in Table 2 were fitted using nonlinear regression to values of the rate constant *k*, estimated from measured rates of reaction from experiments in the packed bed reactor over the temperature range of 500–575 °C for SFO and 475–575 °C for modified samples (as shown in Figure 8, with measured rates used given in the SI, Figure S16), with 95% confidence intervals for each parameter quoted.

**Table 2:** Extracted Kinetic Parameters from Nonlinear Regression of Experimental Measurements in the Packed Bed to eq 2a^a^

sample	*E*_a_ (kJ mol^–1^)	*A* (mol_O_2__ s^–1^ g_OC_^–1^)	*R*^2^ (−)
SFO	102.3 (97.9, 106.7)	22,030 (8,103, 36,320)	0.99
CeO_2_/SFO	66.3 (63.1, 69.5)	152 (81.0, 223)	0.90
(CeO_2_)_ss_SFO	75.7 (73.9, 77.5)	623 (459, 788)	0.98
SCeFO	29.9 (26.7, 33.2)	0.88 (0.46, 1.30)	0.89
Ag/SFO	69.0 (66.0, 71.9)	278 (158, 398)	0.99

a The values in parentheses indicate the estimated 95% confidence intervals for each fitted parameter.

For SFO, SCeFO, and Ag/SFO, no significant difference at the 95% confidence level was found between the parameters estimated from linear and nonlinear fitting (shown in the SI, Section S7, Figure S17, and Table S5). However, a difference between values of kinetic parameters estimated *via* linear and nonlinear regression, outside the 95% confidence intervals, was estimated for CeO_2_/SFO, suggesting a possible substantial contribution from the random error structure to the fitted parameters.^45,46^ Hence, the kinetic parameters for the sample of CeO_2_/SFO should be treated with caution.

As shown in Figure 8, the 95% prediction bands for SFO, (CeO_2_)_ss_SFO, and Ag/SFO did not overlap in the temperature range of 500–575 °C. Hence, the values of *E*_a_ and the values of *A* for SFO, (CeO_2_)_ss_SFO, SCeFO, and Ag/SFO were significantly different at a 95% confidence level. Contrastingly, the difference in values of *E*_a_ and *A* between SFO and CeO_2_/SFO or between CeO_2_/SFO and (CeO_2_)_ss_SFO was found not to be statistically significant. The sample structurally modified with Ce, SCeFO, showed markedly lower values of *E*_a_ and *A* than any other sample. However, since *E*_a_ and *A* are linked, the resulting rates and rate constants are similar to those for other samples, demonstrating that here, in a model-free kinetic parametrization, *E*_a_ should not be interpreted without *A* and, ideally, without the information of the full kinetic triplet of *E*_a_, *A*, and *k*.^51^

Hence, the assumption that all samples follow the same mechanism of oxygen release, allowing for comparable kinetic parameters, might not be valid for SCeFO. For example, given the low porosity of SCeFO observed from SEM images (Figure S7) as compared to that of SFO, oxygen uncoupling from SCeFO might become more readily rate-limited by internal mass transfer rather than the reaction on the surface of OC particles. However, the possible presence of micropores in the SCeFO material not visible under SEM, and/or varying agglomerate size as a result of the milling and calcination procedure used in material preparation, might also contribute to the relatively low reactivity of the SCeFO sample.

The values estimated here for *E*_a_ and *A* for SFO are compared with other values reported in the literature for SrFeO_3−δ_ in the SI, Table S6, with literature values determined using different experimental methodologies (e.g., electrical conductivity relaxation,^52^ TGA,^53^ and packed bed experiments^54^). In particular, the *E*_a_ values reported here for SFO were somewhat lower than those reported previously in the literature (in the range of 128–144 kJ mol^–1^).

All analyses of reaction rates in the packed bed reactor, including determination of kinetic parameters, assume that the reactor behaves isothermally; i.e., at all times during reduction and oxidation, the entirety of the solid and gas in the packed bed is at the set-point of the temperature controller. However, during experiments with gas switching between air and N_2_, some deviation was observed below the set-point during reduction of the OC material (∼5 °C) and above the set-point temperature during reoxidation (up to ∼15 °C for oxidation in air) due to the slow response of the temperature controller as compared to the rapid rates of reactions of the solids. Hence, the decrease in temperature during the endothermic reduction step might result in somewhat underestimated rates of reactions at each temperature set-point, resulting in lower values of *E*_a_ and *A* than might be expected for an isothermal reactor (opposite for reoxidation).

#### 4.1.2 Kinetics of Oxygen Uptake

Reoxidation of OC samples occurred an order of magnitude more rapidly than reduction (Figures 4 and 6); hence, results collected using 0.40 g of OC material for all samples apart from SCeFO could not be used to extract the kinetic parameters of oxidation because of a substantial increase in the reactor temperature above the controller set-point value and the depletion of oxygen in the flowing gas stream.

**Figure 9:**
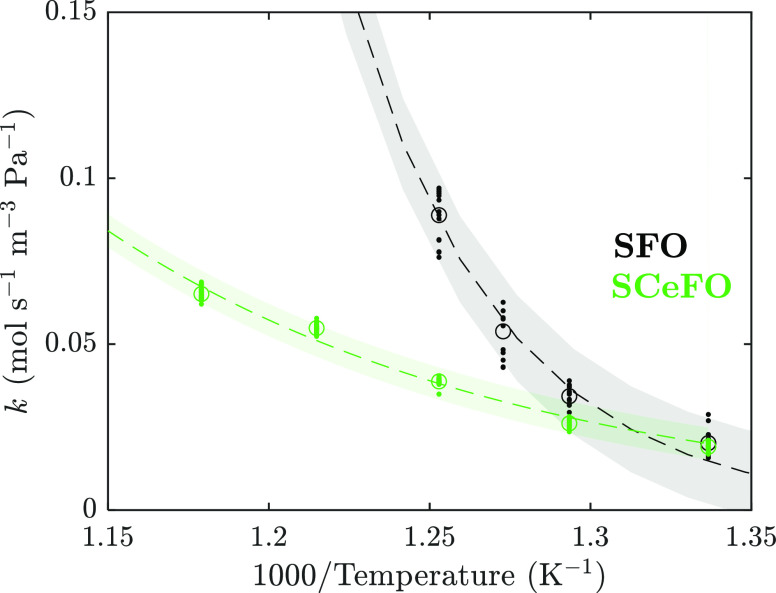
Estimated rate constant, *k*, plotted against inverse temperature for oxidation of SFO in 5.05 vol % oxygen over the range of 475–525 °C and SCeFO in air over the range of 475–575 °C. Points indicate experimental measurements from each cycle, circles indicate average over cycles, dashed lines indicate fitted exponential curves (*R*^2^ = 0.98 for both SFO and SCeFO), and shaded regions indicate 95% confidence intervals.

To mitigate the problem of oxygen depletion, 0.1 g of SFO material (i.e., the sample with the slowest rate of oxidation) was used; nevertheless, measurements above 525 °C could not be used for determination of kinetic parameters for SFO, as a substantial fraction of the oxygen provided in the feed was consumed (shown in the SI, Section S8, Figure S18). Kinetic parameters were, therefore, assessed over the range of 475–525 °C, as shown in Figure 9.

The extracted values of *E*_a_ and *A* for reoxidation of SFO were determined to be *E*_a_ = 177.1 kJ mol^–1^ (with a 95% confidence interval of 163.3–191.0 kJ mol^–1^) and *A* = 3.40 × 10^10^ mol_O_2__ s^–1^ g_OC_^–1^ (with a 95% confidence interval of −3.70 × 10^10^–10.5 × 10^10^ mol_O_2__ s^–1^ g_OC_^–1^). Interestingly, Yoo et al.^52^ reported a little difference in the activation energy between oxidation and reduction of SrFeO_3_ from the measurement of relaxation of electrical conductivity, at 145 ± 0.2 kJ mol^–1^, for small perturbations in equilibrium at pO_2_ = 0.05 atm. Additionally, the value of *E*_a_ estimated here is substantially greater than the activation energy estimated for SFO oxidation in pure O_2_ by Bulfin et al.,^53^ 92 ± 16 kJ mol^–1^, determined from TGA measurements over the temperature range of 177–477 °C.

Curiously, despite showing rapid reduction kinetics, SCeFO showed a relatively slow observed rate of oxygen uptake in air (shown in Figure 9) and hence a limited temperature increase during oxidation, allowing for extraction of kinetic parameters for reoxidation in air over the temperature range of 475–575 °C, at *E*_a_ = 64.0 kJ mol^–1^ (with a 95% confidence interval of 62.6–65.3 kJ mol^–1^) and *A* = 584 mol_O_2__ s^–1^ g_OC_^–1^ (with a 95% confidence interval of 469–706 mol_O_2__ s^–1^ g_OC_^–1^).

For Ag/SFO, reoxidation of the OC material in 5.05 vol % O_2_ was attempted, but the reaction was sufficiently fast that there was no discernable difference between any measurements taken above 500 °C, as oxygen in the gas phase became rapidly depleted (shown in the SI, Section S8, Figure S19).

### 4.2 Mechanistic Considerations

Previous studies investigating modification of perovskite materials with Ag to enhance oxygen transport ascribed the enhanced rate of oxygen transport to a catalytic effect.^23,24^ Particles of silver deposited on the feed site of membranes used for air separation composed of perovskite materials have been found to catalyze the dissociation of molecular O_2_ to lattice oxygen ions with the overall reaction^55^ given in eq 3a. The proposed reaction steps,^56^ indicating the corresponding change in lattice Fe oxidation state as a result of oxygen incorporation, are given in eqs 3bb,c3c.
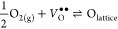
3a
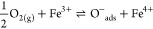
3b
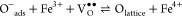
3cThe O_lattice_ species formed are then able to diffuse through the perovskite material and recombine on the permeate side to form pure O_2_. Hence, under conditions where the flow of oxygen from air through a membrane is limited by the rate of incorporation of oxygen into the material of the membrane (i.e., ionic membranes), the overall rate of oxygen transport is increased by the presence of silver. Furthermore, Wang et al.^20^ found that thin films of Ag deposited on the surface of SrFeO_3-δ_ altered the perovskite crystal structure near the Ag–SrFeO_3_ interface by forming a layer of weak Fe–O–Ag bonds with the net transfer of electrons from Ag to SrFeO_3_. The modified structure of the Ag–SrFeO_3_ interface decreased the energy barrier for O^2–^ transport as compared to the bulk SrFeO_3−δ_ perovskite, allowing for the phase transition from the perovskite to brownmillerite to occur at lower temperatures than would be expected thermodynamically for SrFeO_3_.

Here, we find that for partial reduction of SrFeO_3−δ_ (i.e., δ < 0.5, with the material remaining in perovskite form throughout), the presence of Ag on the surface of SrFeO_3−δ_ affects both the rates of oxygen release and reuptake (as shown in Figure 6) and the thermodynamic relationship between pO_2_–*T–*δ at equilibrium of SrFeO_3−δ_ (as shown in Figure 5), with the presence of Ag increasing the concentration of oxygen vacancies at equilibrium under all investigated values of pO_2_. The presence of Ag on the surface of SrFeO_3−δ_ resulted in lower estimated values of (3 – δ_eq_) for Ag/SFO in N_2_ than any reported in the literature for bare SrFeO_3−δ_ in equilbrium with N_2_. Hence, under the experimental conditions investigated here, the role of silver is unlikely to be purely catalytic; rather, the influence of Ag translates to the observed enhancement in the rate of SrFeO_3−δ_ reduction employing both kinetic and thermodynamic effects.

In a similar chemical looping system to the SrFeO_3−δ_ materials impregnated with Ag investigated here, Hwang et al.^57^ found that for reduction and oxidation of Ce_0.5_Zr_0.5_O_2−δ_ impregnated with Pt, the presence of Pt enhanced the rate of oxygen release but had no effect on the rate of oxygen uptake. Hence, the authors concluded that the rate of reduction of Pt/Ce_0.5_Zr_0.5_O_2−δ_ was limited by the surface reaction, i.e., formation of O_2_ molecules from O^2–^ ions, whereas oxidation was limited by O^2–^ diffusion. Here, however, the presence of Ag on SrFeO_3−δ_ increased the rates of both oxygen release and reuptake as compared to unmodified SrFeO_3−δ_, indicating that, under the conditions investigated, both reduction and oxidation of Ag/SFO were affected by the surface reaction up to ∼575 °C, whereas at higher temperatures (600 °C), the rate of reaction was limited by diffusion through the pores (as discussed in the SI, Section S5).

A moderate increase in the rate of oxygen release from SrFeO_3−δ_ was observed for CeO_2_/SFO and (CeO_2_)_ss_SFO. Materials modified with CeO_2_ also showed decreased *T*_onset_ values as compared to SFO. Machida et al.^58,59^ proposed that for composite oxygen carrier materials with CeO_2_ on the surface, CeO_2_ is able to act catalytically in a similar manner to Ag, that is, by facilitating formation of O_2_ molecules from lattice oxygen atoms during reduction, and vice versa during oxidation. Moreover, Tian et al.^18^ found from density functional theory calculations that CeO_2_ deposited on SrFeO_3_ decreased the energy barrier for formation of oxygen vacancies during reduction. Marek et al. also hypothesized that, for (CeO_2_)_ss_SFO, the presence of CeO_2_ helps accelerate the ionic diffusivity of oxygen through the composite material by acting as a “shortcut” through bulk SrFeO_3-δ_.^17^ Given that, here, only a little difference was observed between *T*_onset_ values and kinetic parameters of reduction for CeO_2_/SFO and (CeO_2_)_ss_SFO, it is not possible to conclusively determine which of the proposed mechanisms for enhancement of oxygen release had the greatest influence.

Contrastingly, for SCeFO, a substantial increase in *T*_onset_ was observed as compared to SFO but with a faster rate of oxygen release under CLAS conditions in packed bed experiments. Furthermore, the activation energies and pre-exponential factors estimated for reduction and oxidation of SCeFO were markedly lower than those of SFO. Hence, the rate-limiting step for reduction and oxidation of SCeFO might differ from that of SFO, e.g., resulting from the low porosity of SCeFO and leading to early internal mass transfer effects. Additionally, the 5 atom % Ce doping level in the prepared SCeFO might be near the critical value, reported by Markov and co-workers to be in the range of ∼5–15 atom % Ce,^34^ above which the lattice distortion introduced by the large Ce^4+^ ion to the perovskite crystal partially counterbalances the improvement to oxygen transport induced by partial reduction of Ce^4+^ to Ce^3+^ (and vice versa for reoxidation), resulting in high *T*_onset_ values.

The rates of oxygen release from OC materials modified with Ag and Ce at 500 °C compare favorably with reported values for other candidate materials for CLAS, with CeO_2_/SFO, (CeO_2_)_ss_SFO, SCeFO, and Ag/SFO releasing 95.3, 106.1, 135.9, and 138.9 μmol_O_2__ g_OC_ min^–1^ in packed bed experiments. The rate of oxygen release surpasses Sr_0.8_Ca_0.2_FeO_3−δ_ (70.3 μmol_O_2__ g_OC_ min^–1^), as reported by Li and co-workers;^5,60^ however, none of the materials investigated here released oxygen as rapidly at 500 °C as their Sr_0.8_Ca_0.2_Fe_0.4_Co_0.6_O_3−δ_ (173.1 μmol_O_2__ g_OC_ min^–1^).^5^

In summary, Ag/SFO, CeO_2_/SFO, and (CeO_2_)_ss_SFO were all found to have lower *T*_onset_ values and faster rates of reduction and oxidation than the SFO without any surface, bulk, or structural modification. Future work should investigate optimal loadings of Ag and CeO_2_ and aim to reduce the amount of more expensive dopants. Other noble metal–perovskite combinations are worth studying, as they might show similar or better enhancements than Ag/SFO for oxygen release, improving the energy efficiency of CLAS and enabling novel chemical looping processes. Another possibility, not explored here, but worth further investigation, is incorporating Ag into the bulk of SrFeO_3−δ_ or the perovskite structure.^61^ Both can help gain further control over oxygen release from SrFeO_3−δ_.

## 5 Conclusions

The results from this study demonstrated that materials composed of SrFeO_3−δ_ modified with Ag or CeO_2_, as surface or bulk dopants, donate oxygen faster than the original perovskite. Impregnation of SrFeO_3−δ_ with ∼15 wt % Ag decreased the starting temperature of oxygen uncoupling from 370 C to 310 °C and considerably accelerated the rate of oxygen release. Besides the faster kinetics of oxygen uncoupling, the presence of Ag affected the thermodynamic equilibrium, leading to higher values of δ_eq_ in SrFeO_3−δ_. Surface and bulk modification with CeO_2_ also decreased the *T*_onset_ but only by ∼10–20 °C, slightly increasing the rate of oxygen release. Strontium ferrite, which was structurally doped with Ce to form Sr_0.95_Ce_0.05_FeO_3−δ_, showed a higher *T*_onset_, at around 411 °C, and a slower rate of oxidation in air but a faster rate of reduction in N_2_ than unmodified SrFeO_3−δ_. Kinetic parameters were estimated for reduction of SrFeO_3_-based OC materials modified with Ag, Ce, and CeO_2_ and for oxidation of SrFeO_3−δ_ and Sr_0.95_Ce_0.05_FeO_3-δ_. The marked improvement in the release of oxygen at low temperatures from described modifications of SrFeO_3−δ_ could considerably expand the design space of candidate materials to act as oxygen carriers at relatively mild temperatures (≤500 °C), facilitating development of novel chemical looping processes for selective oxidation.

## Supporting Information Available

Characterization of prepared OC materials, schematic of the packed bed reactor, derivation of rate expression, supplementary TGA results, mass transfer limitations during oxygen release in packed bed experiments, comparison of linear and nonlinear fitting to obtain kinetic parameters, and oxygen depletion over Ag/SFO (PDF)
