# Supplementary material for: Kinetic and Thermodynamic Enhancement of Low-Temperature Oxygen Release from Strontium Ferrite Perovskites Modified with Ag and CeO2
Source: Energy Fuels. 2023 Jun 14;37(13):9487–99. doi: 10.1021/acs.energyfuels.3c01263 (PMC10331733; doi:10.1021/acs.energyfuels.3c01263)
Supplement: Supplementary file 1 — ef3c01263_si_001.pdf [file ef3c01263_si_001.pdf]

Supplementary Information for:

Kinetic and thermodynamic enhancement of low-temperature oxygen release from strontium ferrite perovskites modified with Ag and CeO_2

Alexander R.P. HARRISON[a*], Kien Y. KWONG[a], Yaoyao ZHENG[b], Abhishek BALKRISHNA[a], Alice DYSON[a], Ewa J. MAREK[a*]

[a] *Department of Chemical Engineering and Biotechnology, University of Cambridge, Philippa Fawcett Drive, CB3 0AS, Cambridge, U.K.*

[b] *Department of Engineering, University of Cambridge, Trumpington Street, CB2 1PZ, Cambridge, U.K.*

**Corresponding Authors, arph2@cam.ac.uk, ejm94@cam.ac.uk*

S1. Characterisation of prepared OC materials

X-ray diffraction patterns for the as-prepared OC materials are shown in Fig. S1, with estimated phase compositions given in Table S2, and for spent OC materials after 250 CLAS cycles in the temperature range 500-600°C in Fig. S2. From the XRD measurements, the particles of SFO contained >95 wt% perovskite $SrFeO_3$ phase, with some amount of Ruddlesden-Popper $Sr_3Fe_2O_7$, or unreacted $SrCO_3$, also present. The samples of $(CeO_2)_{ss}$SFO and CeO_2/SFO, produced by solid-state ball milling and wet impregnation, respectively, comprised two distinct phases of $SrFeO_3$ and CeO_2, as previously reported [1]. The estimated loading of CeO_2 in $(CeO_2)_{ss}$SFO from both XRD (Table S2) and ICP (Table S4) measurements was substantially lower than the target value, indicating an error in the preparation, with the achieved CeO_2 content of 2.6 wt% rather than the aimed 4.3 wt%. The XRD pattern for SCeFO showed no

discernible CeO_2 peaks, indicating that the Ce was incorporated into the perovskite structure, rather than forming a separate phase. Instead, a characteristic shift in the $SrFeO_3$ peak at $2\theta = 47.0°$ can be noticed in Fig. S3, in agreement with previous results for SCeFO [2]. The XRD results for Ag/SFO contain additional peaks corresponding to metallic silver, with an estimated Ag loading of 14.5 wt%, in line with the expected value, 15 wt%.

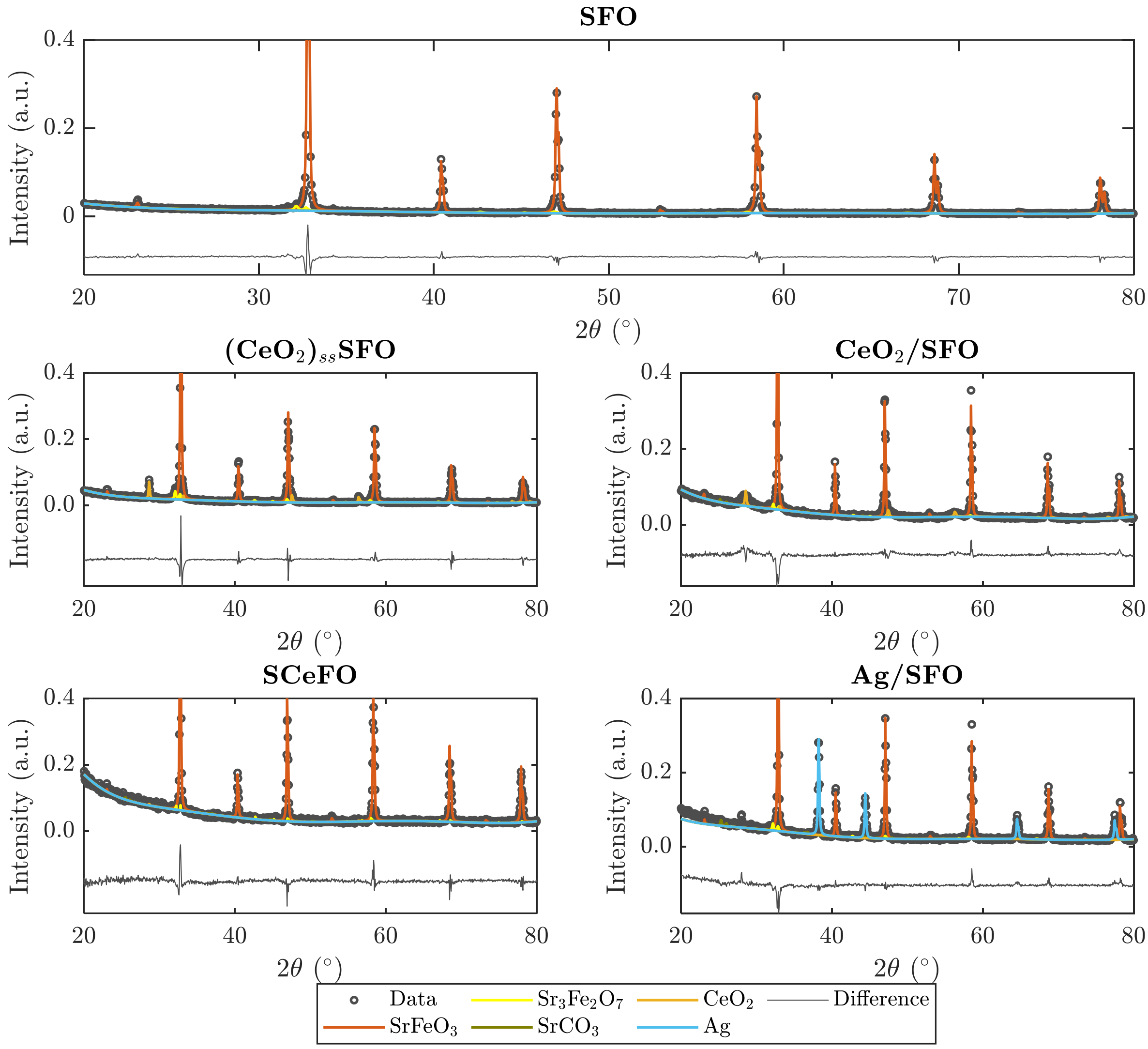

Figure S1: XRD patterns for synthesised materials. Points indicate experimental measurements, lines indicate fitted peaks from reference patterns. SCeFO sample after activation in H_2 and reoxidation (see Section 2.1 of the main manuscript).

Table S1: ICSD database entry codes used for XRD refinement.

Phase	ICSD Collection Code
$SrFeO_3$	91062

SrO	163625
$Sr_3Fe_2O_7$	74437
$SrCO_3$	15195
$SrFeO_{2.5}$	51318
α-Fe_2O_3	56372
CeO_2	24887
Ag	53759

Table S2: Estimated phase composition of synthesised samples from XRD. Fractions of SrO and α-Fe_2O_3 were negligible (<0.1 wt%) for all samples.

Sample	$SrFeO_3$ (wt%)	$Sr_3Fe_2O_7$ (wt%)	$SrCO_3$ (wt%)	CeO_2 (wt%)	Ag (wt%)
SFO	96.0	3.4	0.5	-	-
CeO_2/SFO	91.0	3.0	2.0	3.3	-
$(CeO_2)_{ss}$SFO	90.0	5.9	0.9	2.8	-
SCeFO	95.0	1.4	1.4	0.4	-
Ag/SFO	80.8	2.8	1.9	-	14.5

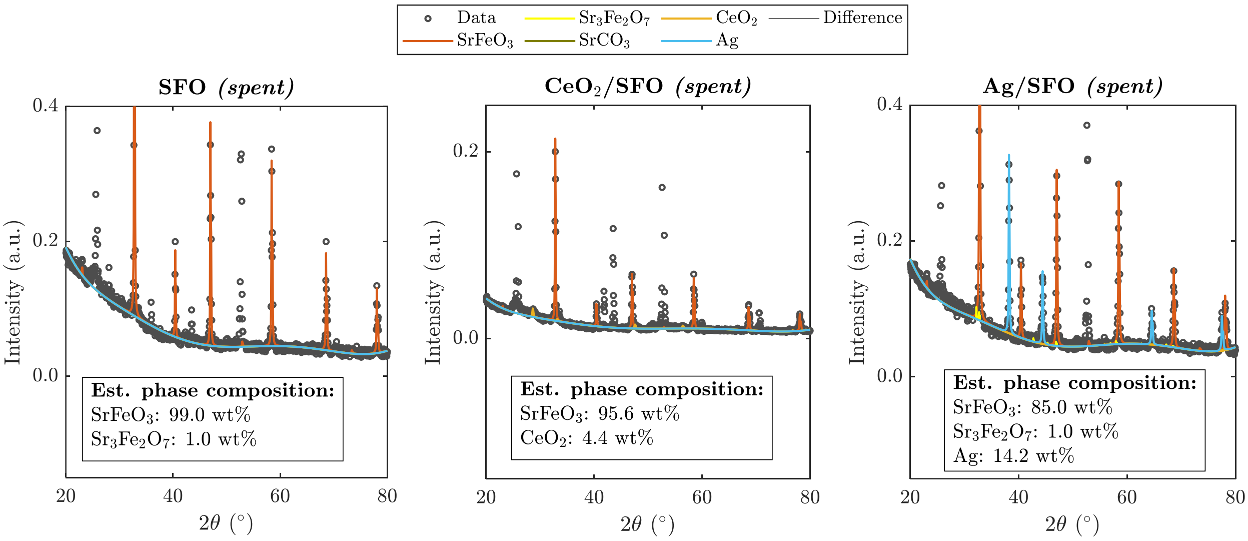

Figure S2: XRD patterns of spent samples of SFO, CeO_2/SFO, and Ag/SFO, showing the material remains predominantly perovskite. Unfitted peaks are ascribed to Al_2O_3 from the experiments in the packed-bed reactor. Estimated compositions were determined by Rietveld refinement, neglecting the mass fraction of Al_2O_3.

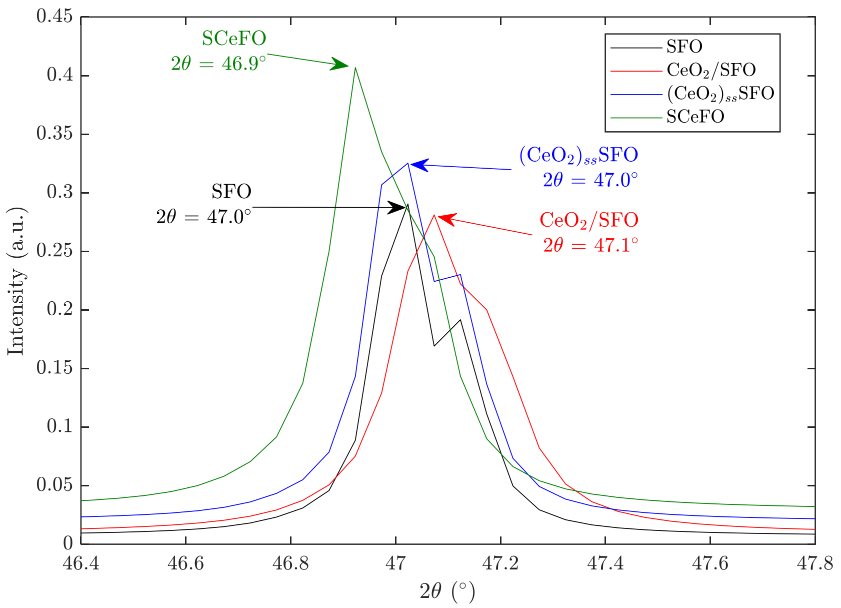

Figure S3: Shift in $SrFeO_3$ peak between samples with un-doped perovskite (SFO, CeO_2/SFO, $(CeO_2)_{ss}$SFO), and the sample with Ce incorporated into the perovskite structure (SCeFO) after activation in H_2 and reoxidation (see Section 2.1 of the main manuscript).

Crystallite size of Ag and CeO_2 in the prepared materials was estimated from XRD measurements, using the Scherrer equation:

$$\tau = \frac{K\lambda}{\beta \cos(\theta)} \qquad \textbf{[Eq. S1]}$$

where τ is the mean crystallite size (nm), K is a dimensionless shape factor (taken as $K \approx 0.9$), λ is the Cu-Kα X-ray wavelength (0.15406 nm), β is the full width at half maximum (radians), and θ is the Bragg angle (radians). Estimated values are given in Table S3, with no change in Ag crystallite when comparing Ag/SFO before and after CLAS cycling, but with spent CeO_2/SFO showing an increase in mean CeO_2 crystallite size after 250 CLAS cycles.

Table S3: Estimated crystallite size of Ag or CeO_2 for Ag/SFO, CeO_2/SFO and $(CeO_2)_{ss}$SFO from XRD peak broadening. 'Spent' samples refer to samples removed from the packed bed reactor after 250 CLAS cycles over the temperature range 500-600°C.

Sample	CeO_2 crystallite size (nm)	Ag crystallite size (nm)
CeO_2/SFO (as-prepared)	6.5 ± 1.0	-
CeO_2/SFO (spent)	11.5 ± 1.2	-
$(CeO_2)_{ss}$SFO	38.8 ± 11.5	-
Ag/SFO (as-prepared)	-	39.4 ± 8.8
Ag/SFO (spent)	-	40.0 ± 18.1

To confirm estimated loadings of CeO_2 on CeO_2/SFO and $(CeO_2)_{ss}$SFO, and Ag on Ag/SFO, ICP-AES measurements were performed, with results summarised in Table S4.

Table S4: Target and measured values of CeO_2 and Ag loadings for surface- and bulk-impregnated samples from ICP-AES measurements.

Sample	Target loading (wt%)	Measured loading (wt%)
CeO_2/SFO	9.3	10.6
$(CeO_2)_{ss}$SFO	4.3	2.5
Ag/SFO	15.0	12.7

Figures S4a and S4b show SEM micrographs of Ag/SFO material before and after 250 CLAS cycles, with little visible change in surface morphology. From measurements of visible Ag particles at the surface of SFO, the size distribution of Ag is given in Fig. S4c. Average particle size of Ag did not change substantially during CLAS cycling (75.0 ± 24 nm before cycling, 74.8 ± 24 nm after cycling) showing that Ag did not sinter or agglomerate over 250 cycles.

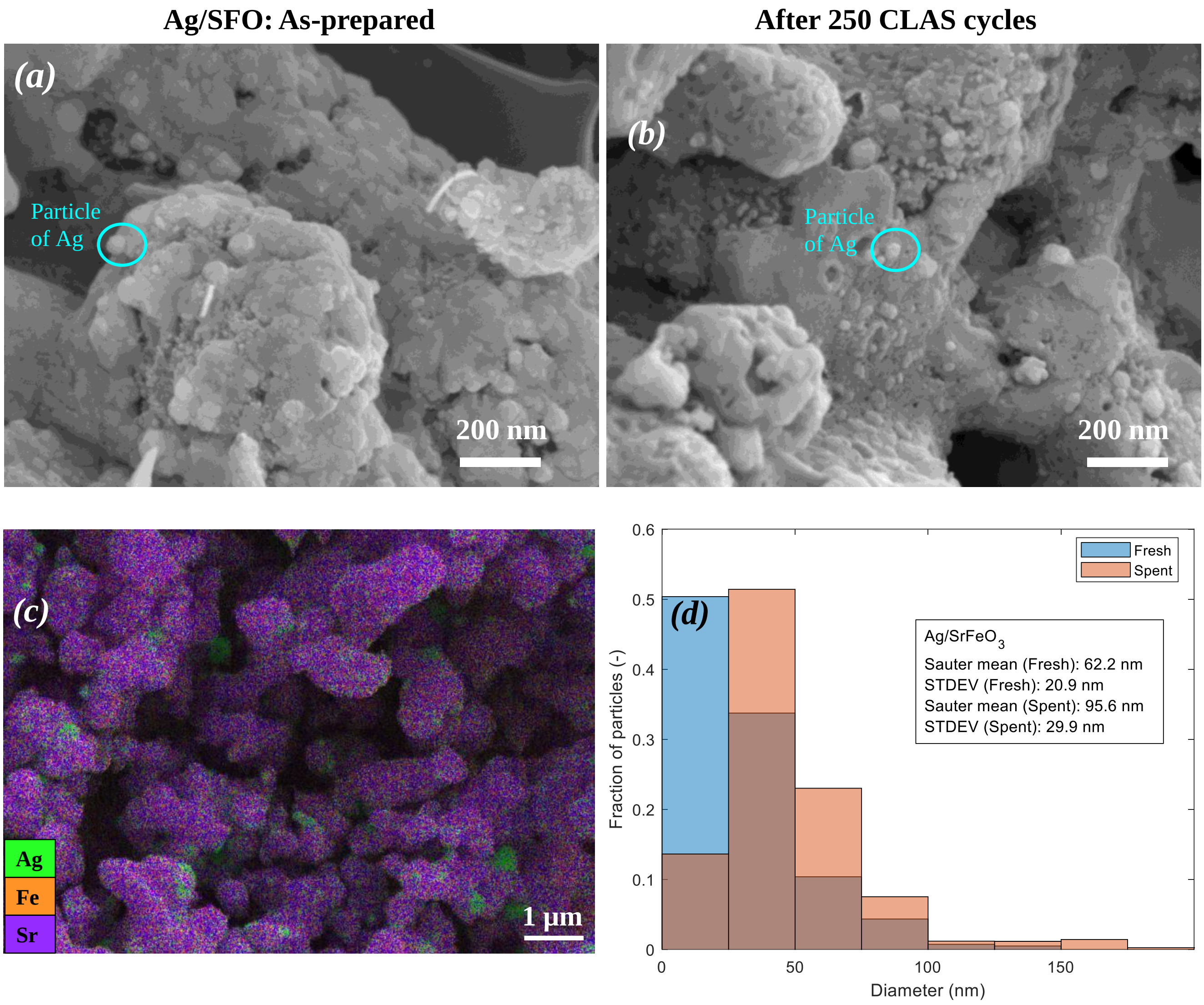

Figure S4: SEM images of Ag/SFO material (a) as-prepared, with particles of Ag highlighted and (b) after 250 CLAS cycles in the temperature range 500-600°C, (c) EDS map showing distribution of Ag, Fe, and Sr at the surface of spent Ag/SFO, and (d) histogram showing particle size distribution of Ag on Ag/SFO, for as-prepared sample (blue bars, 415 particles) and spent sample after 250 cycles of CLAS over the temperature range 500-600°C (orange bars, 426 particles).

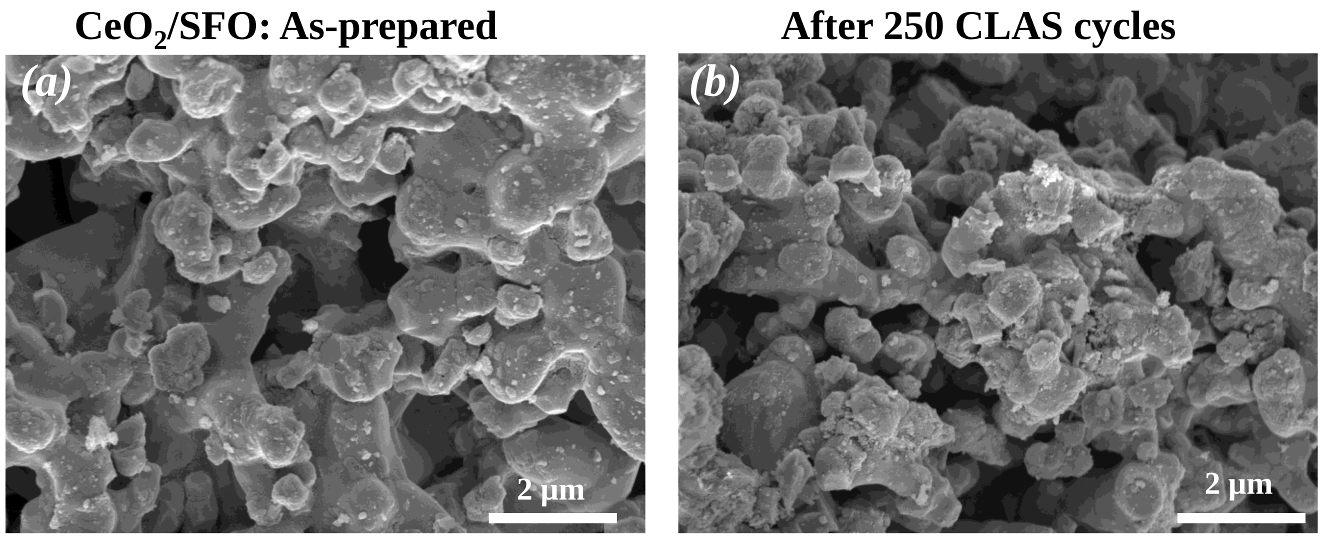

Figure S5: SEM images of CeO_2/SFO material (a) as-prepared and (b) after 250 CLAS cycles in the temperature range 500-600°C.

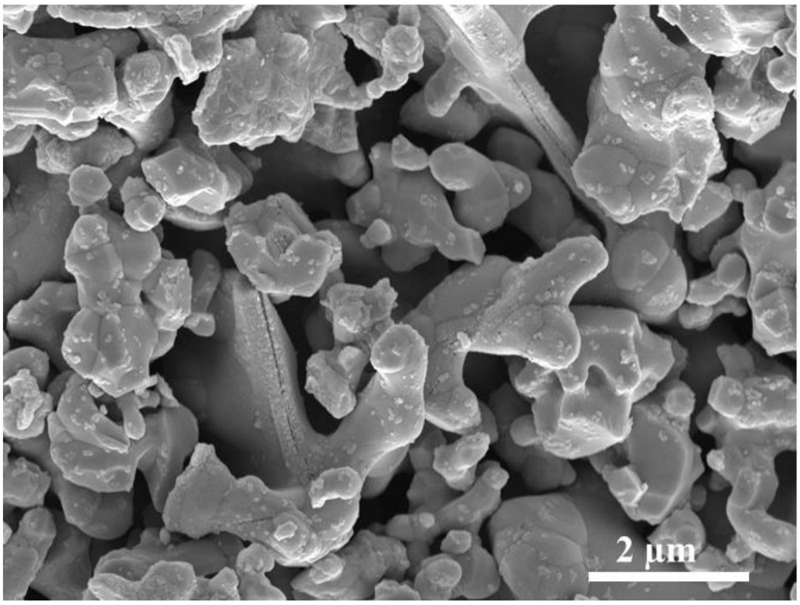

Figure S6: SEM image of as-prepared $(CeO_2)_{ss}$SFO.

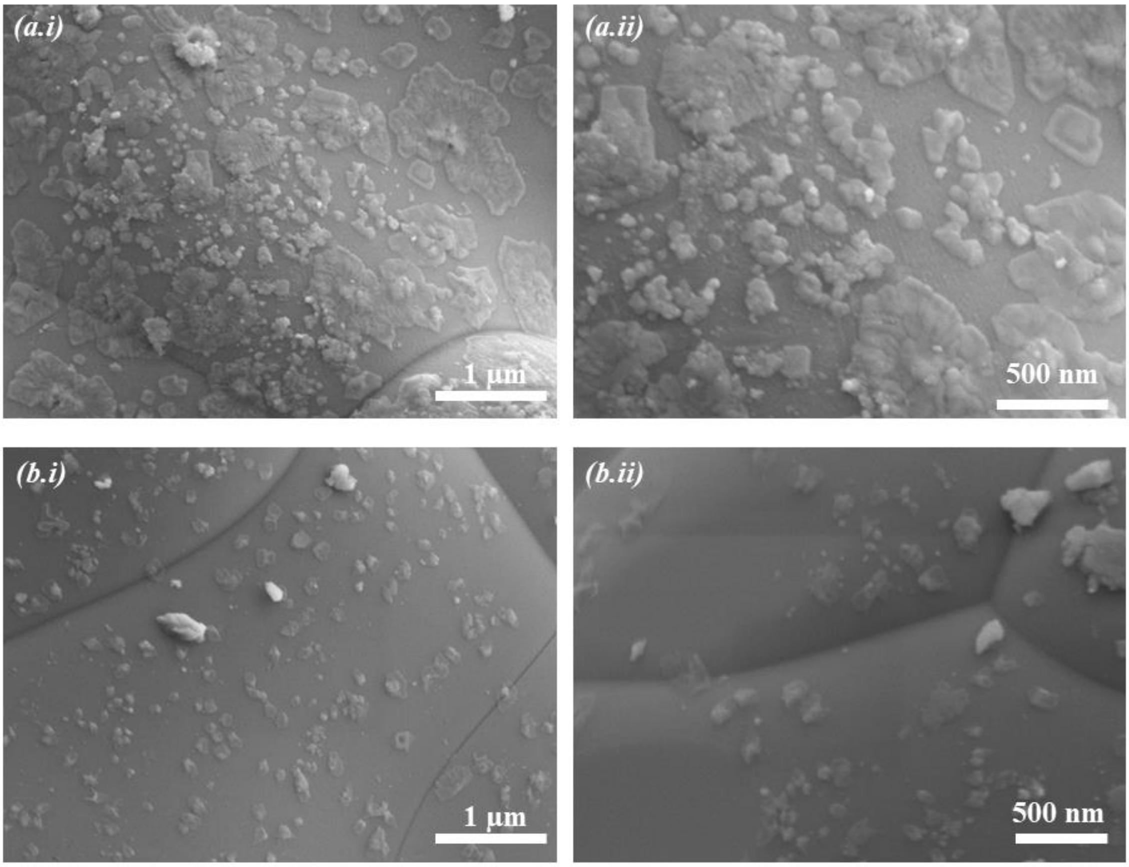

Figure S7: (a) SEM image of SCeFO used in CLAS experiments, i.e. after calcination at 1200°C and activation in H_2, (b) SEM image of fresh SCeFO after calcination at 1200°C.

S2. Schematic of packed-bed reactor

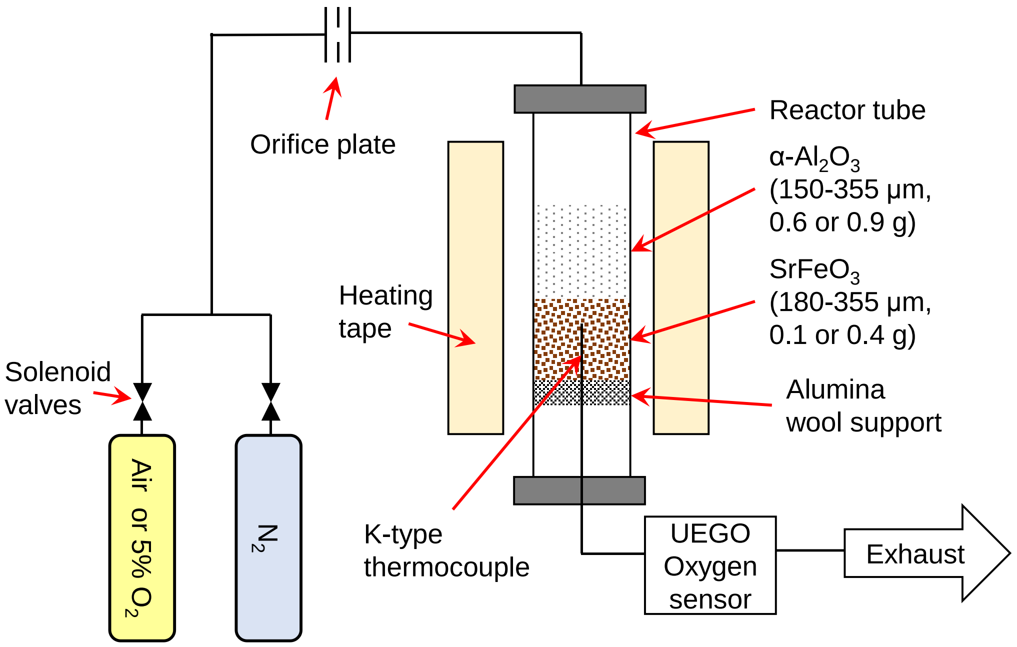

Figure S8: Schematic diagram of packed bed reactor used in experiments.

S3. Derivation of rate expression

Assuming a pseudo-steady state condition and first-order reaction, the concentration profile along the packed bed can be simplified for the case when the reaction is just starting ($t = 0$) and δ is uniform across the bed (*i.e.* no profile in δ has been developed yet, so $\delta = \delta_0$), as explained in [3]. Then, the balance for oxygen gives:

$$\frac{\partial x_{O_2} v}{\partial z} = -kRT(x_{O_2} - x_{O_2,eq}(\delta_0)) \quad \textbf{[Eq. S2]}$$

where x_{O_2} is the mole fraction of oxygen at a point along the bed, $x_{O_2,eq}$ is the mole fraction of oxygen that would be in equilibrium with the solid material, v is the superficial gas velocity through the bed (m s^{-1}), z is the distance along the bed (m), k is the rate constant (mol s^{-1} m^{-3} Pa^{-1}), T is the reaction temperature (K), and R is the molar gas constant (kJ mol^{-1} K^{-1}). The superficial velocity can then be related to the constant molar flux of inert N_2, J_{N_2} (mol m^{-2} s^{-1}):

$$J_{N_2} = v\frac{P}{RT}(1 - x_{O_2}) \quad \textbf{[Eq. S3]}$$

where P is the total pressure in the reactor (taken as ~ 1.01×10^5 Pa).

Combining Eqs. S2 and S3, and integrating over the bed length, L:

$$-\frac{P}{J_{N_2}} k \int_0^L dz = \int_{x_{O_2,in}}^{x_{O_2,out}} \frac{dx_{O_2}}{(1-x_{O_2})^2 (x_{O_2} - x_{O_2,eq}(\delta_0))} \quad \textbf{[Eq. S4]}$$

In the case of a switch in feed gas from air to nitrogen, the value of $x_{O_2,in}$ was taken to be 10^{-5} (*i.e.* using the nominal purity of the cylinder N_2 as 99.999 vol%), the value of $x_{O_2,eq}$ was taken to be 0.21, and the value of $x_{O_2,out}$ was estimated by finding the maximum value of x_{O_2} from the UEGO signal, as shown in Fig. 6 of the main manuscript. Then, by solving the integrals in Eq. S4 analytically, the first-order rate constant, k, is given by

$$k = -\frac{J_{N_2}}{LP}\left[\frac{(x_{O_2}-1)\ln\left(\left|x_{O_2}-x_{O_2,eq}(\delta_0)\right|\right)-\ln(|x_{O_2}-1|)x+\ln(|x_{O_2}-1|)+x_{O_2,eq}(\delta_0)-1}{\left(x_{O_2,eq}(\delta_0)-1\right)^2 (x_{O_2}-1)}\right]_{10^{-5}}^{x_{O_2,max}} \quad \textbf{[Eq. S5]}$$

The apparent reaction constant for re-oxidation of SFO was determined by evaluating Eq. S5 using $x_{in} = 0.0505$ (*i.e.* the mole fraction of oxygen in the supplied gas from the cylinder), $x_{eq} = 10^{-5}$, and $x_{out} = (0.0505 - |x_{UEGO}|)$, where x_{UEGO} is the maximum negative deviation measured using the UEGO oxygen sensor with respect to the blank curve recorded over an inert bed.

S4. Supplementary thermogravimetric analysis (TGA)

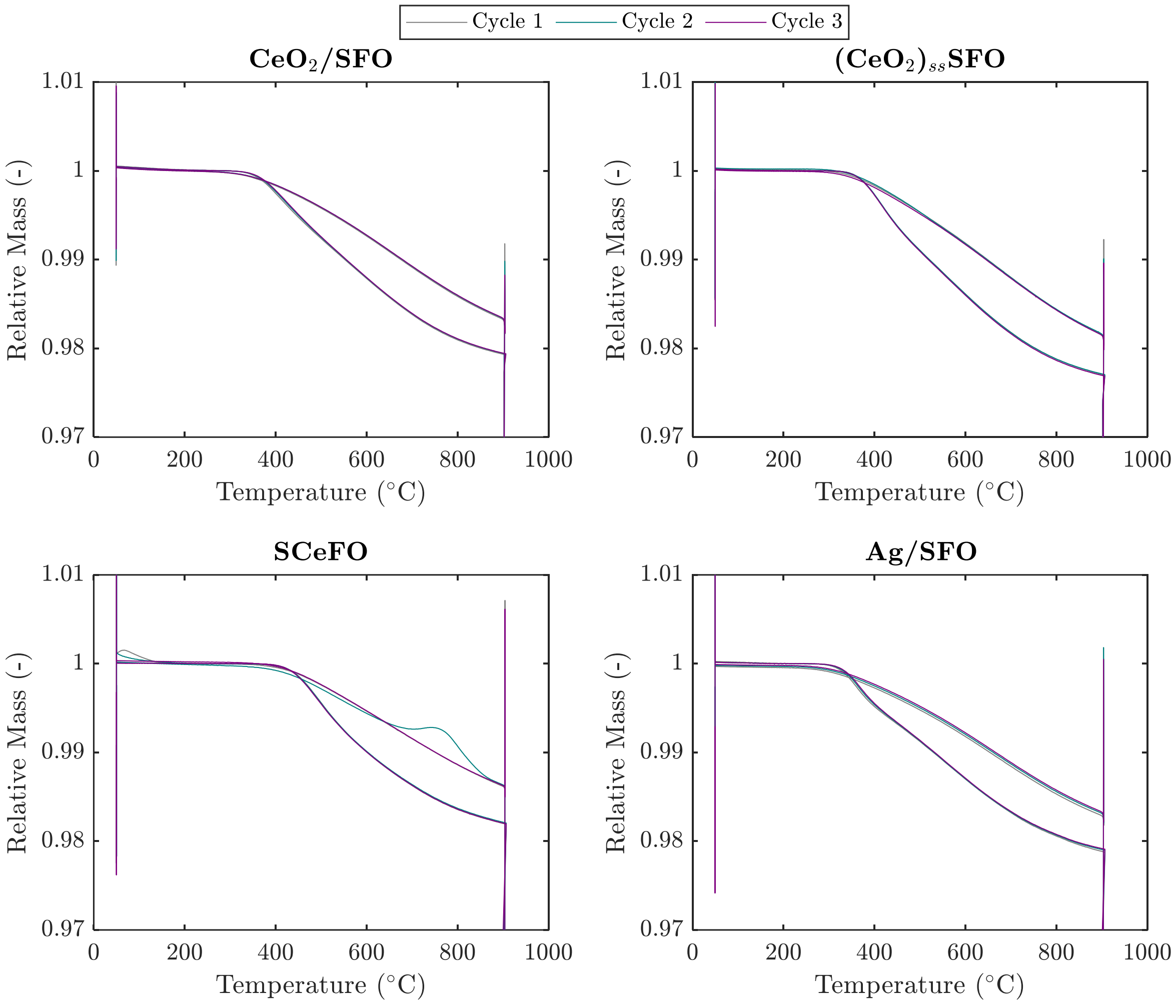

Figure S9: TPR-TPO cycles with reduction in N_2 and oxidation in air for CeO_2/SFO, $(CeO_2)_{ss}$SFO, SCeFO, and Ag/SFO showing minimal difference in mass change between successive cycles for each sample. The deviation observed in cycle 2 for SCeFO at ~800ºC was attributed to a fluctuation in TGA coolant temperature, rather than a genuine change in sample mass.

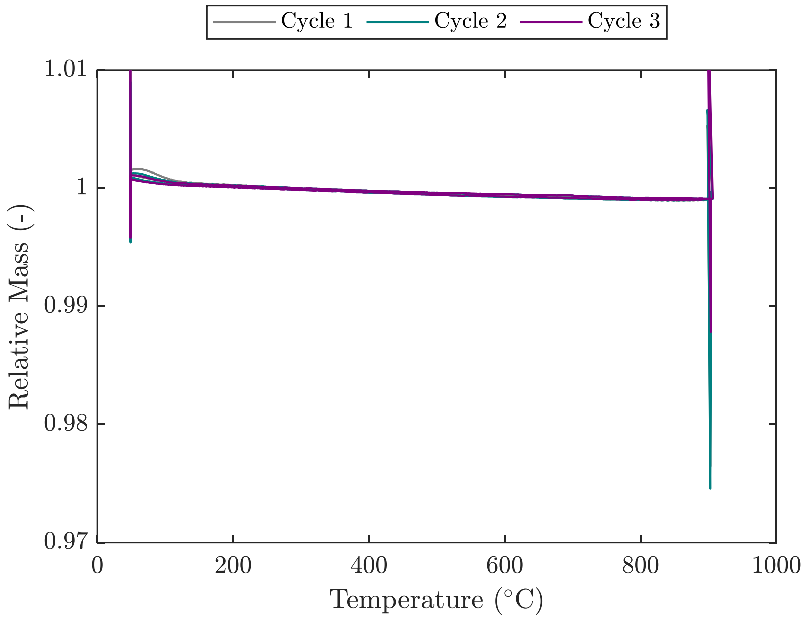

Figure S10: TPR-TPO cycles with reduction in N_2 and oxidation in air for particles of CeO_2, showing a relative mass change of less than 0.01.

Initial stoichiometry of the $SrFeO_{3-\delta}$ perovskite was estimated from the change in gradient associated with the reduction of $SrFeO_{2.5}$ [4,5].

$$M_r(SrFeO_{3-\delta_0}) = M_r(BM) \cdot \frac{1-\lambda_m}{m_{BM}-\lambda_m} \quad \textbf{[Eq. S6a]}$$

$$3 - \delta_0 = \frac{M_r(SrFeO_{3-\delta_0}) - M_r(Sr) - M_r(Fe)}{M_r(O)} \quad \textbf{[Eq. S6b]}$$

where m_{BM} is the relative mass at the change in gradient (as shown on Fig. S11), (3 - δ_0) is the initial oxygen stoichiometry, $M_r(BM)$ is the molar mass of brownmillerite $SrFeO_{2.5}$ (183.465 g mol^{-1}), λ_m is the mass fraction of inert components (*i.e.* wt% of Ag). For Ag/SFO, the first sharp change in gradient during reduction at ~354°C was assumed to correspond to a decrease in rate of reduction at $SrFeO_{2.52}$, as reported by Starkov *et al.* [4] for SFO, whereas the second change in gradient at ~512°C was taken to correspond to the brownmillerite stoichiometry $SrFeO_{2.5}$. For unmodified SFO, no clear change in gradient at $SrFeO_{2.52}$ was observed, potentially because of the relatively high temperature ramp rate (10°C min^{-1}) used here.

From Eq. S6, the initial stoichiometry of the as-prepared SFO and Ag/SFO materials were (3 - δ_0) = 2.82 ± 0.03 and 2.83 ± 0.01 respectively, within the expected range of 2.80-2.85 for $SrFeO_{3-\delta}$ prepared in air [5]. Although the difference in the initial content of oxygen for SFO and Ag/SFO were within experimental uncertainty, the Ag/SFO showed a substantially larger change in mass at low temperatures than SFO, reducing to $SrFeO_{2.52}$ already at ~350°C, see Fig. S11b. The marked increase in rate of reduction of Ag/SFO as compared to SFO at low temperature in H_2 further confirms that Ag enhances oxygen release from SFO.

To confirm that $SrFeO_{2.5}$ stoichiometry was achieved for reduction of Ag/SFO, a sample of Ag/SFO was heated to 500°C in 5% H_2 flow in the TGA, then rapidly quenched (25°C min^{-1} cooling rate) to room temperature in N_2 and characterised *via* XRD (shown in Fig. S11c), confirming that $SrFeO_{2.5}$ was the main phase present.

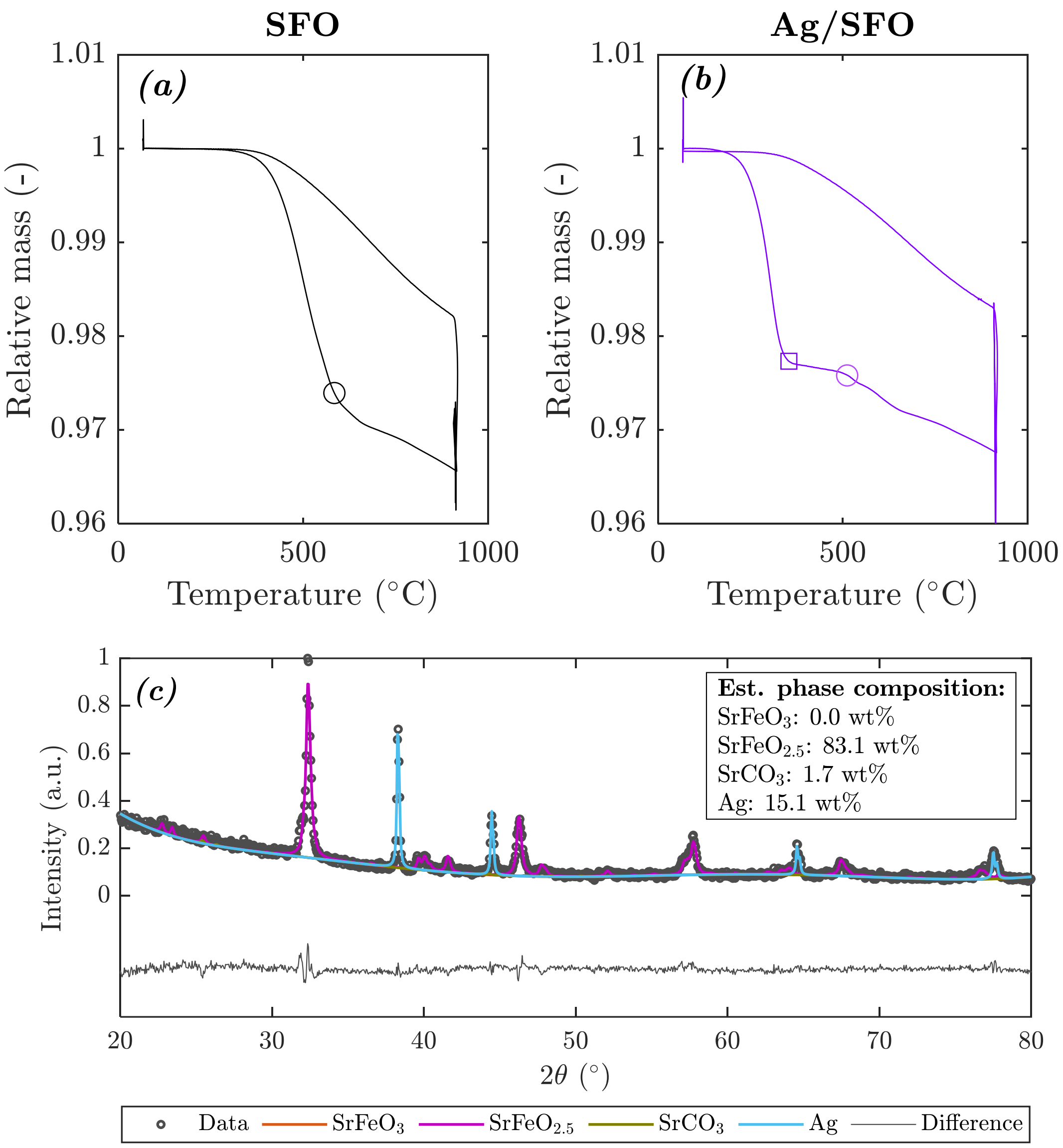

Figure S11: Reduction and oxidation in 5% hydrogen and air respectively of (a) SFO and (b) Ag/SFO, and (c) XRD pattern of Ag/SFO heated to 500°C in 5% H_2, then rapidly quenched, with estimated phase composition from Rietveld refinement. The 2nd of three redox cycles is shown for both samples, with circles indicating the change in gradient associated with $SrFeO_{2.5}$ stoichiometry. For Ag/SFO, the point on the reduction curve corresponding to an estimated stoichiometry of $SrFeO_{2.52}$ is indicated by a square.

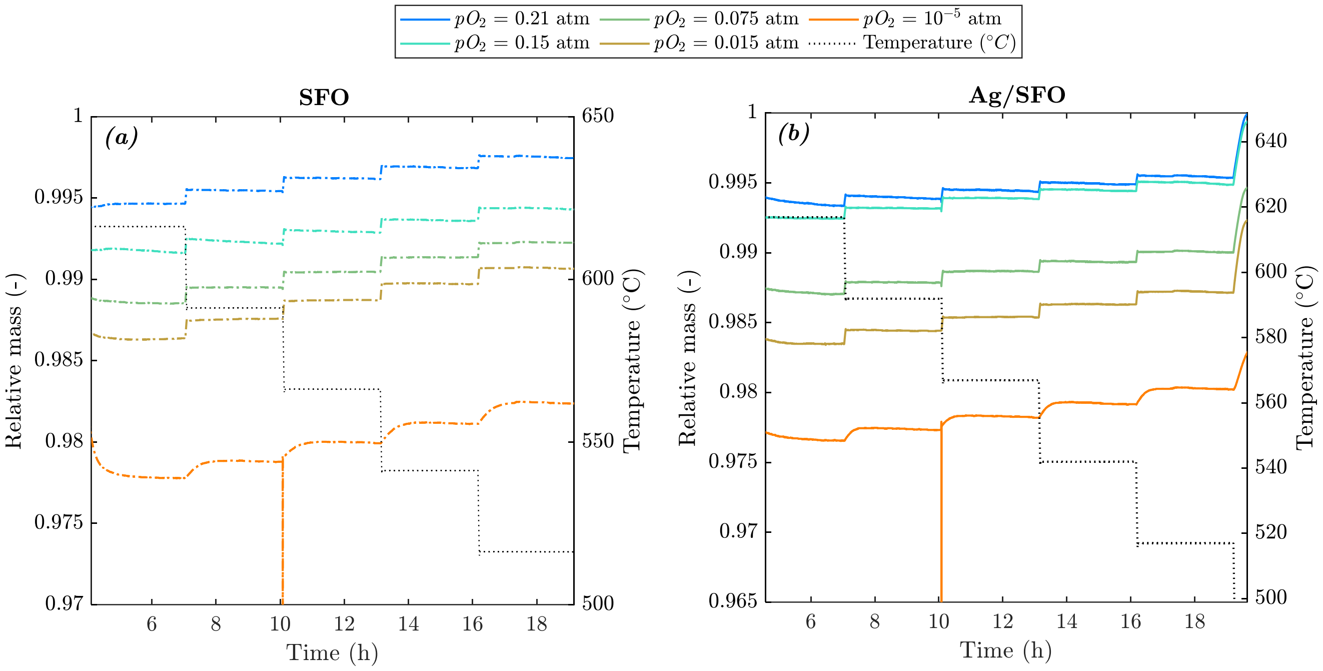

Figure S12: TGA curves for (a) SFO (dash-dot lines) and (b) Ag/SFO (solid lines), brought to equilibrium over the temperature range 500-600°C in varied pO_2 *atmospheres.*

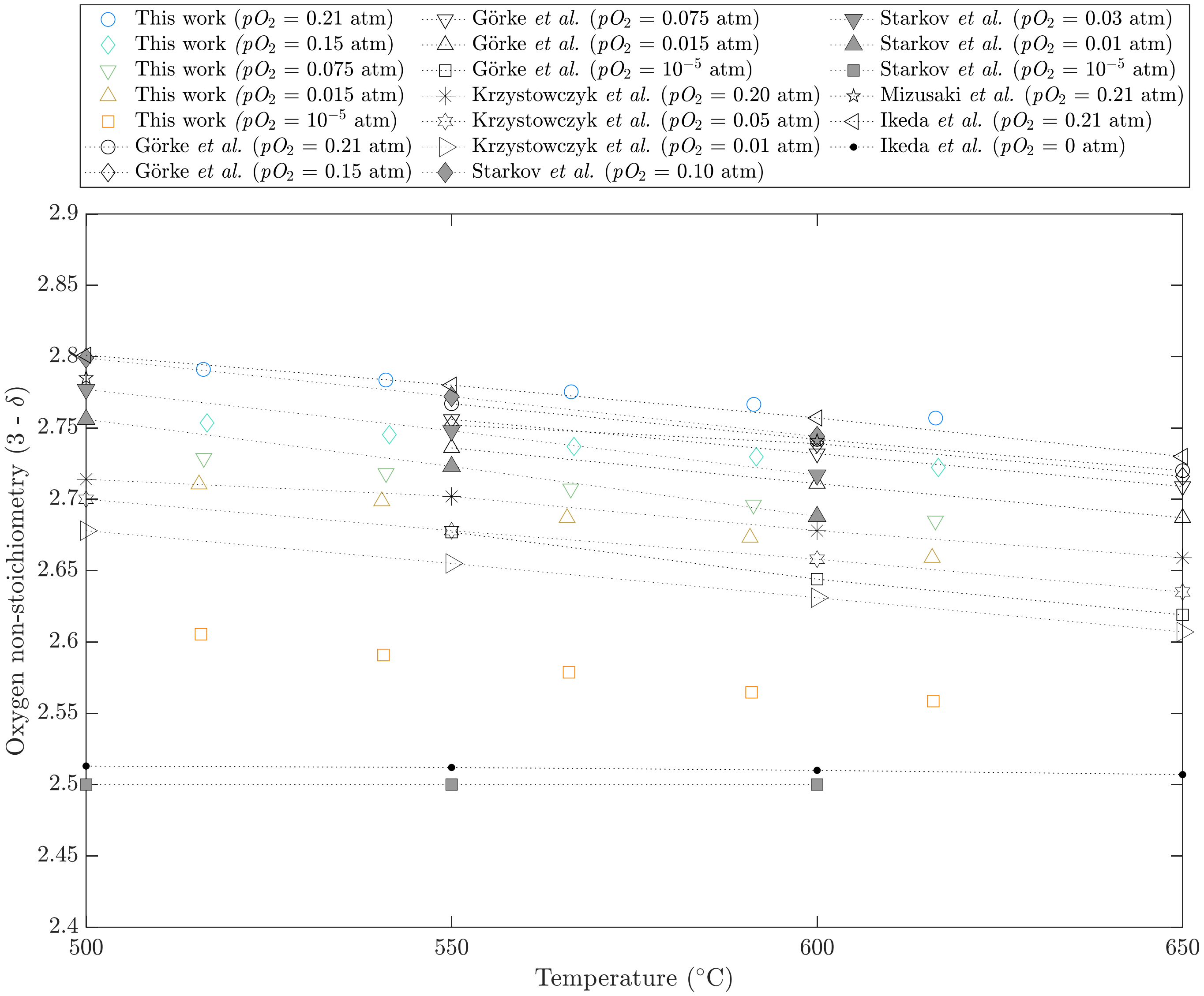

Figure S13: Comparison between obtained equilibrium non-stoichiometry values for SFO with published values reported by Görke et al. [3], *Starkov* et al. [4], *Krzystowczyk* et al. [6], *Mizusaki* et al. [7], *and Ikeda* et al. [8], *with dotted lines included between points to aid interpretation.*

S5. Mass transfer limitations on oxygen release in packed bed experiments

During experiments in a packed bed reactor, the measured rate of release of oxygen might be limited by (1) external mass transport between the surface of the particles of OC material and the flowing gas stream, (2) internal mass transport within the pores of particles of OC materials, (3) thermodynamic equilibrium.

To confirm that the experimental arrangement used to estimate kinetics of oxygen release and uptake would not be limited by external mass transfer from the solids to the gas phase, the maximum rate of mass transport from the surface of the OC particles to the gas stream was estimated. Assuming that the rate of oxygen transport is approximately constant across a thin packed-bed at the start of the particle reduction step, the rate of oxygen transport can be assessed as:

$$r = \frac{k_{M,g} A_{MT}}{\rho_s V(1-\varepsilon)} \frac{P}{RT} \left(x_{O_2} - x_{eq} \right) \qquad \textbf{[Eq. S7]}$$

where r is the rate of oxygen transport *per* mass of oxygen carrier (mol s^{-1}), $k_{M,g}$ is the external mass transfer coefficient (m s^{-1}), A_{MT} is the area of particles available for mass transfer (m^2), V_p is the volume of packed bed (m^3), ε is the bed voidage (taken as ~0.4), ρ_s is the density of the oxygen carrier (kg m^{-3}), P is the total pressure of the system (Pa), T is the gas temperature (K), and x_{O2} and x_{eq} correspond to the mole fractions of oxygen in the gas stream and at equilibrium with the OC material, respectively. The mass transfer coefficient was then estimated from the correlation of Wakao and Funazkri [9]

$$Sh = 2 + 1.1 Sc^{0.33} Re^{0.6} \qquad \textbf{[Eq. S8]}$$

where *Sh* is the Sherwood number, *Sc* is the Schmidt number for the gas phase, and *Re* is the Reynolds number. To evaluate *Sh* and *Re,* the characteristic length

$$d_c = \langle d_p \rangle \cdot \left(\frac{\varepsilon}{1-\varepsilon} \right) \qquad \textbf{[Eq. S9]}$$

was used, where $\langle d_p \rangle$ is the geometric mean of the range of particle sizes used in the packed bed (*i.e.* $\langle d_p \rangle = \sqrt{180 \cdot 300}$ = 253 μm for the packed beds of OC particles used here).

Evaluating Eq. S7 over the temperature range 500-600°C, the estimated rate of oxygen transport at the start of the reduction experiments was ~ 50 mol s^{-1} g_{OC}, *i.e.* 6 orders of magnitude greater than the rates of reduction and 5 orders of magnitude greater than the maximum rate of oxidation measured from experiments.

At high rates of reaction, the observed rate of oxygen release from the OCs may be limited by internal mass transfer (*i.e.* diffusion within pores of the OC particles). Internal diffusion is characterised by the Thiele modulus, ϕ , given for a first-order reversible reaction by Bischoff [10] as

$$\phi^2 = \frac{k_v L^2 g(c)}{2\int_{c_{O_2,eq}}^{c_{O_2,bulk}} D_{eff}\, g(c)\, dc} \qquad \textbf{[Eq. S10a]}$$

where k_v is the rate constant for reaction (s^{-1}), D_{eff} (m^2 s^{-1}) is the effective diffusivity of oxygen within the pores, L is a characteristic length (m), $c_{O_2,eq}$ is the concentration of oxygen in equilibrium with the OC material (mol m^{-3}), $c_{O_2,bulk}$ is the bulk concentration of oxygen in the gas phase (mol m^{-3}), and *g(c)* is a function describing the dependence of the reaction rate on oxygen concentration, given here for a first-order reversible reaction by

$$g(c) = \left(c_{O_2,eq} - c_{O_2,local}\right) \qquad \textbf{[Eq. S10b]}$$

$$r_{v,obs} = k_v g(c) \eta_{eff} \qquad \textbf{[Eq. S10c]}$$

where $r_{v,obs}$ is the observed rate of reaction *per* particle in the packed bed (mol s^{-1} m^{-3}) (positive values of $r_{v,obs}$ correspond to oxygen release, and negative values of $r_{v,obs}$ correspond to oxygen uptake), and η_{eff} is an effectiveness factor. The effectiveness factor is taken as for a reversible first order reaction, which was shown by Bohn to give the same dependency on ϕ as an irreversible first order reaction[11]:

$$\eta_{eff} = \frac{3}{\phi^2}(\phi\, coth(\phi) - 1) \qquad \textbf{[Eq. S10d]}$$

We note, however, that the oxygen release from a solid oxide involves a zero-order forward reaction and a first order backward reaction (if expressed per mol of O_2), possibly affecting the resulting dependency of $\eta_{eff}(\phi)$.

In the case where the influence of external mass transport of gas away from the surface of the particle is negligible, as assumed here, $c_{O_2,local} \approx c_{O_2,bulk}$, where $c_{O_2,bulk}$ is the overall concentration of oxygen in the bulk gas stream.

Effective diffusivity of oxygen within the pores is then given by

$$D_{eff} = \frac{\varepsilon_p}{\tau_p} D_m \qquad \textbf{[Eq. S11a]}$$

where D_m is the molecular diffusivity of oxygen in nitrogen ($m^2\, s^{-1}$), estimated using Chapman-Enskog theory [12], ε_p is the porosity of the OC materials, and τ_p is the pore tortuosity (taken as $\tau_p = 2$). Porosity was estimated from

$$\varepsilon_p = 1 - \frac{\rho_s}{\rho_{SrFeO_3}} \qquad \textbf{[Eq. S11b]}$$

where ρ_s is the density of particles of SFO (taken as $\rho_s = 2486$ kg m^{-3}), and ρ_{SrFeO_3} is the density of non-porous $SrFeO_3$, taken to be $\rho_{SrFeO_3} = 5570$ kg m^{-3} from the Springer Materials database [13]. The rate of reaction *per* particle was estimated from the measured overall rate of reaction measured over the entire bed volume, r (mol s^{-1} m^{-3}) using

$$r_{v,obs} = r\frac{\pi \langle d_p \rangle^3}{6V(1-\varepsilon)} \qquad \textbf{[Eq. S12]}$$

For each observed initial rate of reaction from experiments in the packed-bed, values of ϕ, k_v, and η_{eff} were found by solving Eqs. S10a-c, taking $\eta_{eff} = 1$ as an initial guess, then iterating until k_v, ϕ and η_{eff} converged. The values of ϕ estimated for oxygen release and re-uptake in the packed bed are shown in Fig. S15.

Internal mass transfer limitation may be neglected if the criterion [14]

$$\phi^2 < 0.603 \qquad \textbf{[Eq. S13]}$$

is satisfied. Hence, for each temperature investigated, the critical value of *r* such that both sides of Eq. S13 are equal (*i.e.* the threshold for internal mass transfer contributing significantly to observed rate of reaction) was calculated. As shown in Fig. S14a, for reduction of SFO, CeO_2/SFO, $(CeO_2)_{ss}$SFO, and Ag/SFO, measurements at ≤575°C were below the threshold for internal mass transfer limitation. For SCeFO, the porosity from SEM images was markedly lower than the other samples (shown in Figs. S4-S8), and so SCeFO was excluded from calculations of Thiele modulus.

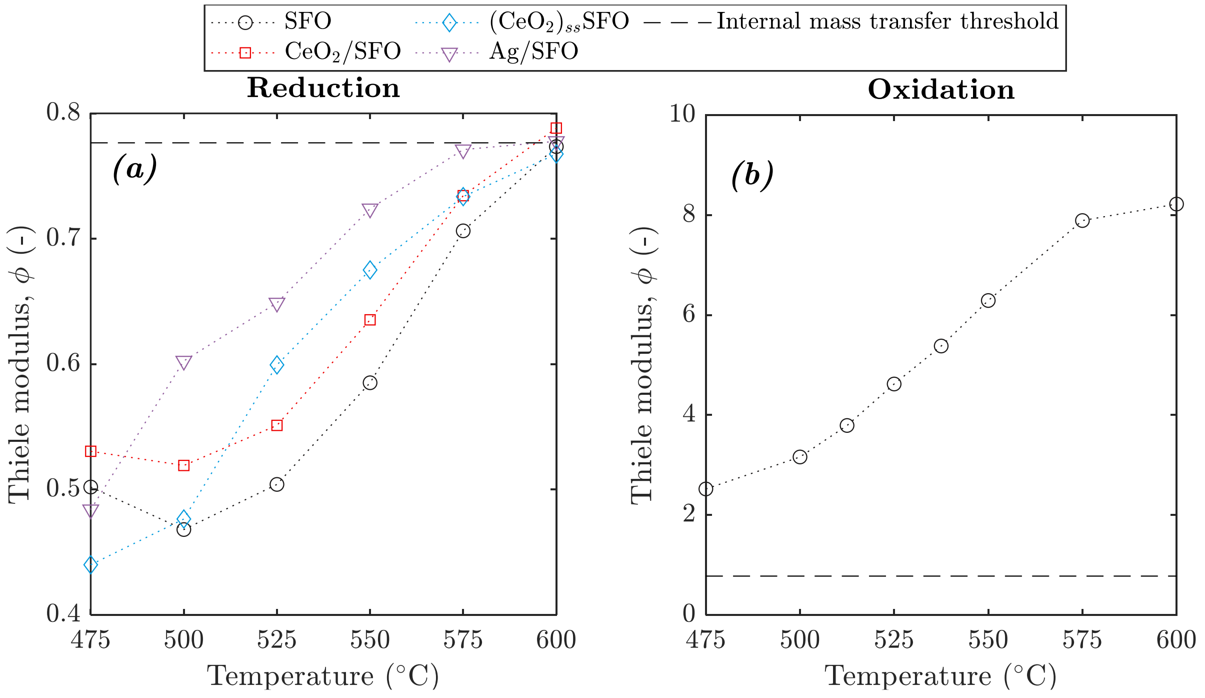

Figure S14: Thiele modulus, ϕ, estimated for (a) oxygen release, and (b) oxygen uptake with dashed lines indicating threshold below which internal mass transfer limitation was estimated to be negligible, from Eq. S13.

For re-oxidation of SFO, shown in Fig. S14b, the estimated values of ϕ substantially exceed the criterion specified in Eq. S13; hence, the observed rate of reaction was likely limited by internal mass transfer. To account for this, the estimated values of the rate constant from Eq. S5, *k'*,

were divided by an effectiveness factor, η_{eff}, given as a function of ϕ for spherical particles by [15].

$$k = \frac{k'}{\eta_{eff}} \quad \textbf{[Eq. S14a]}$$

$$\eta_{eff} = \frac{3}{\phi^2}(\phi \coth(\phi) - 1) \quad \textbf{[Eq. S14b]}$$

As ϕ is an implicit function of k, Eq. S14 was solved iteratively until values of k, ϕ, and η_{eff} converged. Final values of η_{eff} were in the range 0.73-0.40 over the temperature range 475-550°C, with the values of k determined from Eq. S14 used in subsequent calculations of activation energy and pre-exponential factor.

Rate of oxygen release can also be limited by thermodynamic equilibrium, as described by Görke *et al.* [3]. The extent of equilibrium limitation is described by the dimensionless group $\frac{kRTL}{v}$, *i.e.* the ratio of the speed of the reaction front through the packed bed to the superficial velocity of gas through the bed, where k is the first order rate constant (mol s^{-1} m^{-3} Pa^{-1}), v is the superficial velocity of the gas (m s^{-1}), and L is the length of the active bed (m). If $\frac{kRTL}{v} \ll 1$, no significant profile in δ develops along the bed, if $\frac{kRTL}{v} \sim 1$ a profile develops but remains away from equilibrium, and as $\frac{kRTL}{v} \to \infty$, the value of $x_{O_2} \to x_{O_2,eq}(\delta)$. The estimated values of $\frac{kRTL}{v}$ for reduction and oxidation of samples investigated are given in Fig. S15.

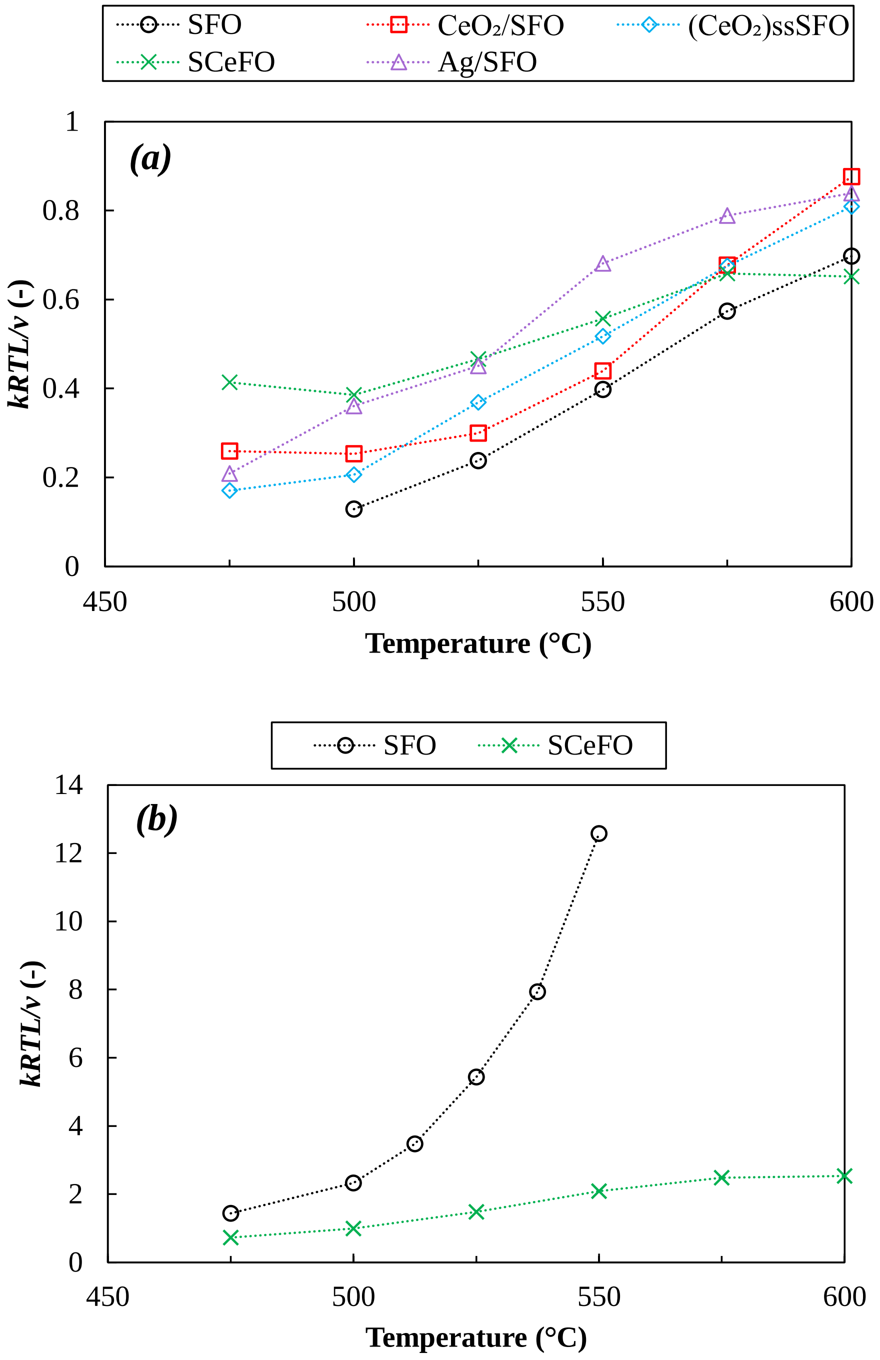

Figure S15: Estimates of kRTL/v *for (a) reduction and (b) re-oxidation.*

For all measurements of reduction of the OC materials, shown in Fig. S15a, $\frac{kRTL}{v} < 1$, indicating little influence of equilibrium limitation on rate of oxygen release. However, for reoxidation of SFO, and SCeFO, shown in Fig. S15b, $\frac{kRTL}{v} > 1$ for all temperatures investigated, indicating that the rate of oxygen uptake was affected by thermodynamic equilibrium. Hence, the values

of k estimated for oxidation of SFO and SCeFO are likely lower than would be observed in the case of kinetic limitation only.

S6. Comparison of linear and non-linear kinetic fitting

Kinetic parameters were estimated from gas-cycling experiments by assuming that when the maximum rate of reaction is observed (approximately 0.07 s after the inlet gas is switched from air to N_2), the material in the bed is approximately in equilibrium with air (*i.e.* pO_2 = 0.21 atm). Connecting the measured rates of reaction to δ in oxygen non-stoichiometry, requires further assumption that δ can be approximated as δ_0. Apparent activation energy, E_a, and pre-exponential factor, A, were then extracted from the measured values for the maximum rates by using linear and non-linear regression [16,17]. For all samples of interest, the rate of oxygen release was affected by internal mass-transfer at 600°C (as described in Fig. S14).

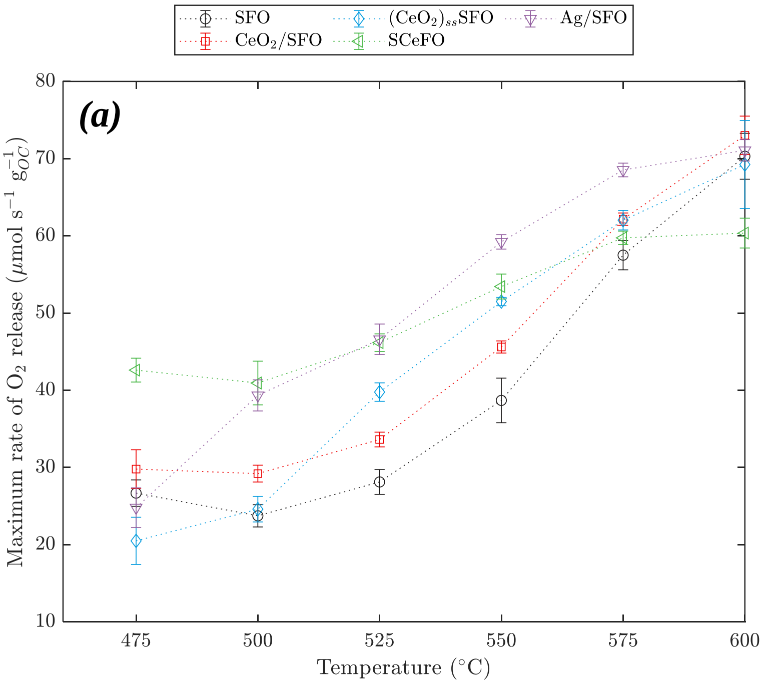

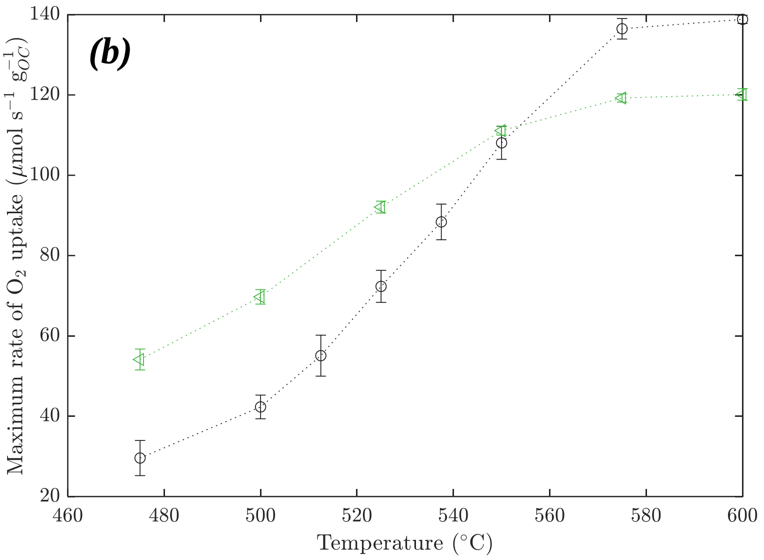

Figure S16: Maximum observed rates of reaction for samples undergoing (a) reduction and (b) oxidation. Points indicate average over repeated redox cycles, error bars indicate standard deviation from 46-49 cycles.

At 475°C in Fig. 16 a, SFO showed slow oxygen release, close to the experimental uncertainty, whereas for samples modified with CeO_2, Ce, or Ag, appreciable rates of oxygen release were estimated at 475°C. All samples showed insufficient oxygen release at 450°C for measurements to fitted to estimate E_a and A. Hence, measurements were fitted over the temperature range 500-575°C for SFO, and over the range 475-575 C for all other samples. Given that each measured temperature point consisted of multiple repeated cycles, results from all cycles were included when fitting regression parameters. Representative non-linear and linear fits are shown in Fig. S17. The extracted values of E_a and A *via* non-linear and linear regression are summarised in Table S5.

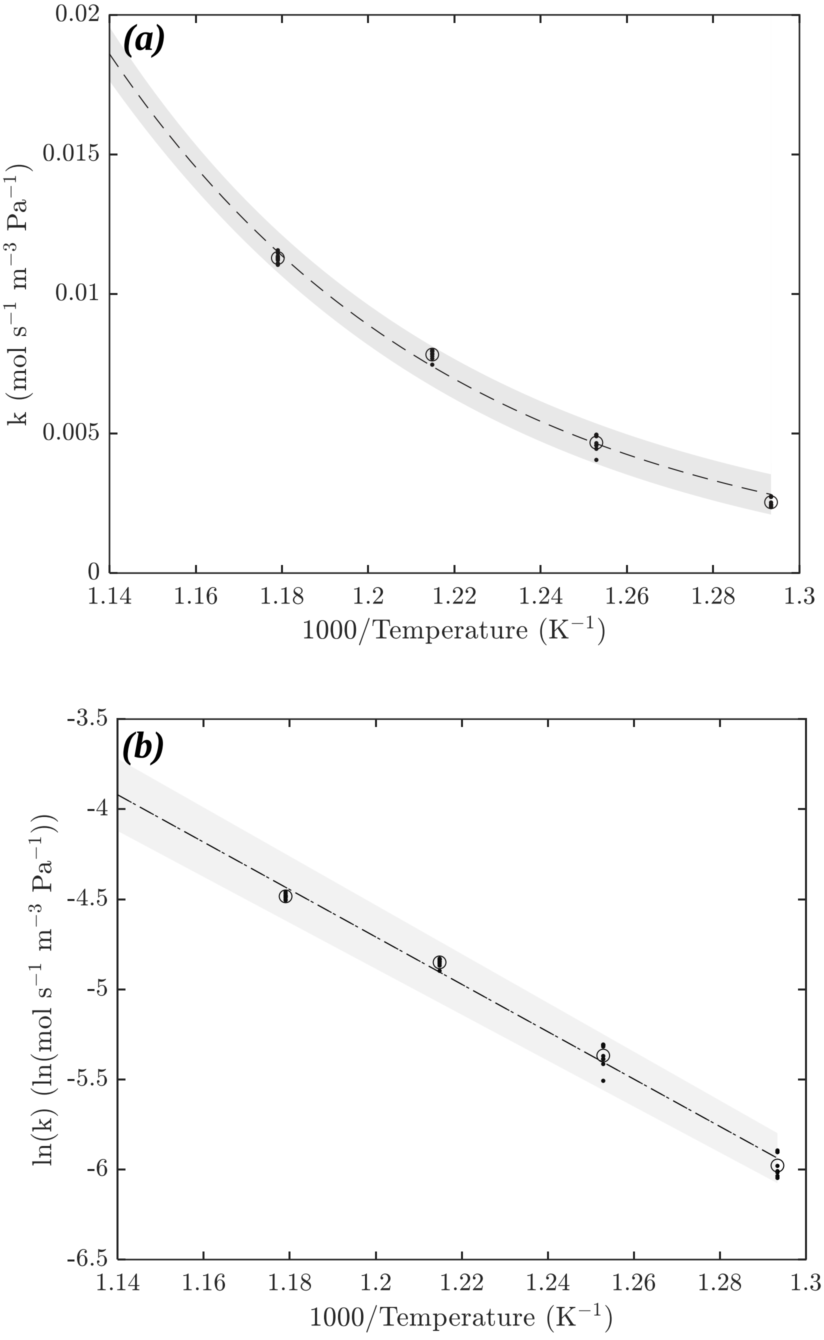

Figure S17: (a) Non-linear and (b) linear fit for oxygen release from SFO. Points indicate experimental measurements, circles indicate average value at each temperature point, dashed line indicates fitted curve, shaded area indicates 95% prediction band for fitted parameters.

Table S5: Extracted kinetic parameters from linear and non-linear regression of experimental measurements in the packed bed. Bracketed values indicate estimated 95% confidence intervals for each fitted parameter.

Sample	E_a (kJ mol^{-1}) (Linear)	A ($\mathrm{mol_{O_2} s^{-1} m^{-3} Pa^{-1}}$) (Linear)	R^2 (-) (Linear)	E_a (kJ mol^{-1}) (Non-linear)	A ($\mathrm{mol_{O_2} s^{-1} m^{-3} Pa^{-1}}$) (Non-linear)	R^2 (-) (Non-linear)
SFO	109.3 *(104.9, 113.6)*	66170 *(32860, 120600)*	0.99	102.3 *(97.9, 106.7)*	22,030 *(8,103, 36,320)*	0.99
CeO_2/SFO	51.5 *(48.5, 54.6)*	16.9 *(10.6, 26.9)*	0.83	66.3 *(63.1, 69.5)*	152 *(81.0, 223)*	0.90
$(CeO_2)_{ss}$SFO	77.9 *(75.8, 80.0)*	862 *(625, 1187)*	0.95	75.7 *(73.9, 77.5)*	623 *(459, 788)*	0.98
SCeFO	26.8 *(25.1, 28.6)*	0.54 *(0.41, 0.70)*	0.81	29.9 *(26.7, 33.2)*	0.88 *(0.46, 1.30)*	0.89
Ag/SFO	68.4 *(66.6, 70.2)*	267.1 *(203.5, 350.6)*	0.96	69.0 *(66.0, 71.9)*	278 *(158, 398)*	0.99

A significant difference (outside 95% confidence intervals) between estimated values of E_a and A was estimated for the sample of CeO_2/SFO, indicating a possible substantial contribution of random errors to the estimated parameters [16]. Hence, the fitted kinetic parameters for CeO_2/SFO should be treated with caution.

Table S6: Comparison of estimated apparent kinetic parameters with literature values. Bracketed values indicate estimated 95% confidence band for fitted parameters; uncertainty values for literature values are reported where available.

Experimental method	Temperature range (°C)	E_a (kJ mol^{-1})	A ($\mathrm{mol_{O_2} s^{-1} m^{-3} Pa^{-1}}$)	Reference(s)
Packed-bed reactor	500-575	102.3 *(97.9, 106.7)*	22030 *(8103, 36320)*	This work
Packed-bed reactor	500-600	128.9 ± 4.7	388000	[3,18]
Thermogravimetric analysis	177-477	144 ± 16	243000	[19]
Packed-bed reactor	500-900	135	Not reported	[20]
Electrical conductivity relaxation	790-1000	128 ± 0.6	Not reported	[21]

S7. Oxygen depletion over Ag/SFO

In experiments using dilute (5.05 vol%) O_2 to re-oxidise oxygen carrier materials, the high reactivity of Ag/SFO resulted in total depletion of the oxygen in the gas stream. A comparison between the oxygen depletion profiles for SFO, and Ag/SFO, is shown in Figs. S18 and S19, showing the rate of re-oxidation of Ag/SFO was sufficient to fully deplete the available oxygen, for all experiments at or above 525°C. Contrastingly, for unmodified SFO, the full depletion of available oxygen was only observed for measurements at or above 575°C.

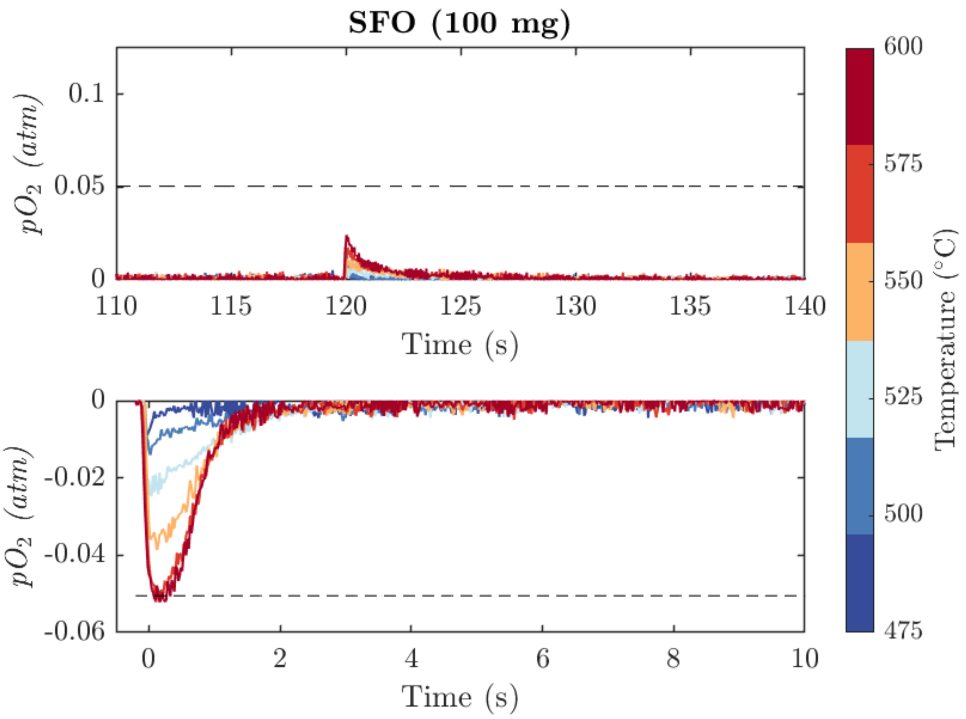

Figure S18: Graphs showing oxygen release and re-uptake for 0.10 g of SFO, reduced under N_2 for 120 s, and re-oxidised in 5.05vol% O_2 for 120s. Dashed line corresponds to pO_2 = 0.05, indicating consumption of all available oxygen.

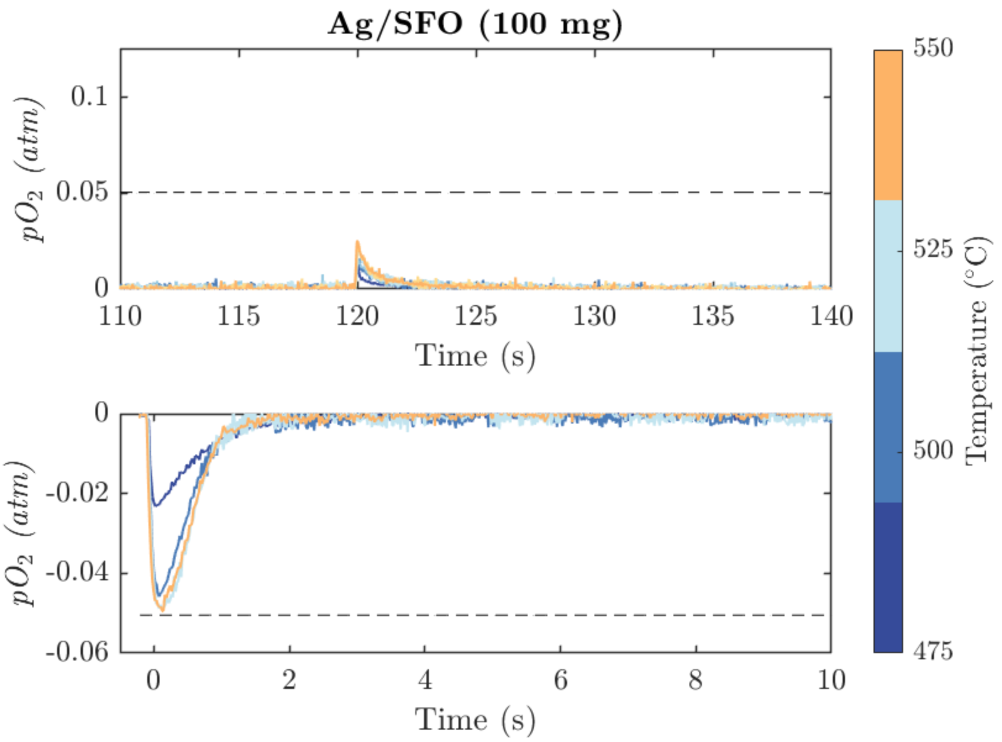

Figure S19: Graphs showing oxygen release and re-uptake for 0.10 g of Ag/SFO, reduced under N_2 for 120 s, and re-oxidised in 5.05vol% O_2 for 120s. Dashed line corresponds to pO_2 *= 0.05, indicating consumption of all available oxygen.*

Supplementary References

(1) Marek, E. J.; Gabra, S.; Dennis, J. S.; Scott, S. A. High Selectivity Epoxidation of Ethylene in Chemical Looping Setup. *Applied Catalysis B: Environmental* **2020**, *262* (October 2019), 118216. https://doi.org/10.1016/j.apcatb.2019.118216.

(2) Deganello, F.; Liotta, L. F.; Longo, A.; Casaletto, M. P.; Scopelliti, M. Cerium Effect on the Phase Structure, Phase Stability and Redox Properties of Ce-Doped Strontium Ferrates. *Journal of Solid State Chemistry* **2006**, *179* (11), 3406–3419. https://doi.org/10.1016/j.jssc.2006.06.027.

(3) Görke, R. H.; Marek, E. J.; Donat, F.; Scott, S. A. Reduction and Oxidation Behavior of Strontium Perovskites for Chemical Looping Air Separation. *International Journal of Greenhouse Gas Control* **2020**, *94*, 102891. https://doi.org/10.1016/j.ijggc.2019.102891.

(4) Starkov, I.; Bychkov, S.; Matvienko, A.; Nemudry, A. Oxygen Release Technique as a Method for the Determination of "δ–PO 2 –T" Diagrams for MIEC Oxides. *Phys. Chem. Chem. Phys.* **2014**, *16* (12), 5527–5535. https://doi.org/10.1039/C3CP52143E.

(5) Marek, E.; Hu, W.; Gaultois, M.; Grey, C. P.; Scott, S. A. The Use of Strontium Ferrite in Chemical Looping Systems. *Applied Energy* **2018**, *223* (November 2017), 369–382. https://doi.org/10.1016/j.apenergy.2018.04.090.

(6) Krzystowczyk, E.; Wang, X.; Dou, J.; Haribal, V.; Li, F. Substituted $SrFeO_3$ as Robust Oxygen Sorbents for Thermochemical Air Separation: Correlating Redox Performance with Compositional and Structural Properties. *Physical Chemistry Chemical Physics* **2020**, *22* (16), 8924–8932. https://doi.org/10.1039/D0CP00275E.

(7) Mizusaki, J.; Okayasu, M.; Yamauchi, S.; Fueki, K. Nonstoichiometry and Phase Relationship of the SrFeO2.5SrFeO3 System at High Temperature. *Journal of Solid State Chemistry* **1992**, *99* (1), 166–172. https://doi.org/10.1016/0022-4596(92)90301-B.

(8) Ikeda, H.; Nikata, S.; Hirakawa, E.; Tsuchida, A.; Miura, N. Oxygen Sorption/Desorption Behavior and Crystal Structural Change for SrFeO3−δ. *Chemical Engineering Science* **2016**, *147*, 166–172. https://doi.org/10.1016/J.CES.2016.03.034.

(9) Wakao, N.; Funazkri, T. Effect of Fluid Dispersion Coefficients on Particle-to-Fluid Mass Transfer Coefficients in Packed Beds: Correlation of Sherwood Numbers. *Chemical Engineering Science* **1978**, *33* (10), 1375–1384. https://doi.org/10.1016/0009-2509(78)85120-3.

(10) Bischoff, K. B. An Extension of the General Criterion for Importance of Pore Diffusion with Chemical Reactions. *Chemical Engineering Science* **1967**, *22* (4), 525–530. https://doi.org/10.1016/0009-2509(67)80035-6.

(11) Bohn, C. D. The Production of Pure Hydrogen with Simultaneous Capture of Carbon Dioxide. Ph.D, thesis, University of Cambridge, Cambridge, 2010.

(12) Poling, B. E.; Prausnitz, J. M.; O'connell, J. P. *The Properties of Gases and Liquids*, 5th ed.; McGraw Hill: New York, 2001. https://doi.org/10.1036/0070116822.

(13) Villars, P.; Cenzual, K. *SrFeO3 Crystal Structure: Datasheet from "PAULING FILE Multinaries Edition – 2012."* SpringerMaterials. https://materials.springer.com/isp/crystallographic/docs/sd_1503839 (accessed 2023-02-16).

(14) Vannice, M. A. *Kinetics of Catalytic Reactions*; Springer US: Boston, MA, 2005. https://doi.org/10.1007/b136380.

(15) Fogler, H. S. *Elements of Chemical Reaction Engineering*, 5th ed.; Prentice Hall: Philadelphia, 2016.

(16) Barrie, P. J. The Mathematical Origins of the Kinetic Compensation Effect: 1. the Effect of Random Experimental Errors. *Physical Chemistry Chemical Physics* **2011**, *14* (1), 318–326. https://doi.org/10.1039/C1CP22666E.

(17) Barrie, P. J. The Mathematical Origins of the Kinetic Compensation Effect: 2. the Effect of Systematic Errors. *Physical Chemistry Chemical Physics* **2011**, *14* (1), 327–336. https://doi.org/10.1039/C1CP22667C.

(18) Görke, R. H. Evaluating New Oxygen Transfer Materials for Air Separation, Oxy-Fuel Combustion and Other Processes (PhD Thesis), University of Cambridge, 2018. https://doi.org/10.17863/CAM.47887.

(19) Bulfin, B.; Vieten, J.; Richter, S.; Naik, J. M.; Patzke, G. R.; Roeb, M.; Sattler, C.; Steinfeld, A. Isothermal Relaxation Kinetics for the Reduction and Oxidation of SrFeO3 Based Perovskites. *Physical Chemistry Chemical Physics* **2020**, *22* (4), 2466–2474. https://doi.org/10.1039/c9cp05771d.

(20) Bychkov, S. F.; Popov, M. P.; Nemudry, A. P. Study of the Oxygen Exchange Kinetics in the Nonstoichiometric Oxide SrFeO3–δ under Isostoichiometric Conditions Using the Oxygen Partial Pressure Relaxation Technique. *Kinetics and Catalysis* **2016**, *57* (5), 697–703. https://doi.org/10.1134/S0023158416050050.

(21) Yoo, J.; Verma, A.; Wang, S.; Jacobson, A. J. Oxygen Transport Kinetics in SrFeO[Sub 3−δ], La[Sub 0.5]Sr[Sub 0.5]FeO[Sub 3−δ], and La[Sub 0.2]Sr[Sub 0.8]Cr[Sub 0.2]Fe[Sub 0.8]O[Sub 3−δ] Measured by Electrical Conductivity Relaxation. *Journal of The Electrochemical Society* **2005**, *152* (3), A497. https://doi.org/10.1149/1.1854617.
